# Transcriptomic-based evaluation of trichloroethylene glutathione and cysteine conjugates demonstrate phenotype-dependent stress responses in a panel of human in vitro models

**DOI:** 10.1007/s00204-022-03436-6

**Published:** 2022-12-28

**Authors:** Liliana Capinha, Yaran Zhang, Anna-Katharina Holzer, Anna-Katharina Ückert, Melinda Zana, Giada Carta, Cormac Murphy, Jenna Baldovini, Zahra Mazidi, Johannes Grillari, Andras Dinnyes, Bob van de Water, Marcel Leist, Jan N. M. Commandeur, Paul Jennings

**Affiliations:** 1 Division of Molecular and Computational Toxicology, Amsterdam Institute for Molecules, Medicines and Systems, Vrije Universiteit Amsterdam, De Boelelaan 1108, 1081 HZ Amsterdam, The Netherlands; 2 Genomics of Neurodegenerative Diseases and Aging, Human Genetics, Vrije Universiteit Amsterdam, Amsterdam UMC Location VUmc, Amsterdam, The Netherlands; 3 In Vitro Toxicology and Biomedicine, Dept Inaugurated By the Doerenkamp-Zbinden Foundation, University of Konstanz, 78457 Constance, Germany; 4 BioTalentum Ltd, Godollo, 2100 Hungary; 5 Evercyte GmbH, Vienna, Austria; 6 Department of Physiology and Animal Health, Hungarian University of Agriculture and Life Sciences, Institute of Physiology and Animal Nutrition, Gödöllő, 2100 Hungary; 7 Division of Drug Discovery and Safety, Leiden Academic Centre for Drug Research (LACDR), Leiden University, Leiden, The Netherlands; 8 Department of Biotechnology, Institute of Molecular Biotechnology, BOKU - University of Natural Resource and Life Science (BOKU), Vienna, Austria; 9 Ludwig Boltzmann Institute for Traumatology, The Research Center in Cooperation with AUVA, Vienna, Austria

**Keywords:** Cytotoxicity, Transcriptomics, Glutathione conjugation pathway, Multi-organ toxicity, Hazard identification

## Abstract

Environmental or occupational exposure of humans to trichloroethylene (TCE) has been associated with different extrahepatic toxic effects, including nephrotoxicity and neurotoxicity. Bioactivation of TCE via the glutathione (GSH) conjugation pathway has been proposed as underlying mechanism, although only few mechanistic studies have used cell models of human origin. In this study, six human derived cell models were evaluated as in vitro models representing potential target tissues of TCE-conjugates: RPTEC/TERT1 (kidney), HepaRG (liver), HUVEC/TERT2 (vascular endothelial), LUHMES (neuronal, dopaminergic), human induced pluripotent stem cells (hiPSC) derived peripheral neurons (UKN5) and hiPSC-derived differentiated brain cortical cultures containing all subtypes of neurons and astrocytes (BCC42). A high throughput transcriptomic screening, utilizing mRNA templated oligo-sequencing (TempO-Seq), was used to study transcriptomic effects after exposure to TCE-conjugates. Cells were exposed to a wide range of concentrations of S-(1,2-*trans*-dichlorovinyl)glutathione (1,2-DCVG), S-(1,2-*trans*-dichlorovinyl)-L-cysteine (1,2-DCVC), S-(2,2-dichlorovinyl)glutathione (2,2-DCVG), and S-(2,2-dichlorovinyl)-L-cysteine (2,2-DCVC). 1,2-DCVC caused stress responses belonging to the *Nrf2 pathway* and *Unfolded protein response* in all the tested models but to different extents. The renal model was the most sensitive model to both 1,2-DCVC and 1,2-DCVG, with an early *Nrf2*-response at 3 µM and hundreds of differentially expressed genes at higher concentrations. Exposure to 2,2-DCVG and 2,2-DCVC also resulted in the upregulation of *Nrf2 pathway* genes in RPTEC/TERT1 although at higher concentrations. Of the three neuronal models, both the LUHMES and BCC42 showed significant *Nrf2*-responses and at higher concentration UPR-responses, supporting recent hypotheses that 1,2-DCVC may be involved in neurotoxic effects of TCE. The cell models with the highest expression of γ-glutamyltransferase (GGT) enzymes, showed cellular responses to both 1,2-DCVG and 1,2-DCVC. Little to no effects were found in the neuronal models from 1,2-DCVG exposure due to their low GGT-expression. This study expands our knowledge on tissue specificity of TCE S-conjugates and emphasizes the value of human cell models together with transcriptomics for such mechanistic studies.

**Supplementary Information:**

The online version contains supplementary material available at 10.1007/s00204-022-03436-6.

## Introduction

In the last two decades the development of new strategies to entirely phase out animal testing on chemical hazard information and risk assessment has highly increased. Legislations in the European Union (EU) have enforced the “Three Rs” policy by the requirement to Replace, Reduce and Refine the use of animals whenever possible (European Directive 2010/63/EU). Developing and improving new approach methodologies (NAMs) is considered crucial for implementing these tools as primary testing approaches for hazard identification that can be eventually applied for regulatory purposes (Knight et al. 2021). Many progress has been made in the development of human-relevant in vitro methodologies to study the toxicodynamics of chemicals (Escher et al. 2019; Krebs et al. 2020). Transcriptomics is considered an extremely valuable tool for detecting and understanding early cellular responses underlying target organ toxicity. The recent developments in transcriptomics technology have increased efficiency while decreasing costs allowing high throughput studies in multiple cell systems and/or concentration ranges (van der Stel et al. 2020; Harrill et al. 2021). In the present study, we utilized a high throughput templated oligo transcriptomic assay with sequencing read-out named TempO-Seq (Limonciel et al. 2018). This technique does not require RNA purification, is able to discriminate highly homologous gene families and has demonstrated excellent reproducibility (Limonciel et al. 2018; Lee et al. 2021).

**Fig. 1:**
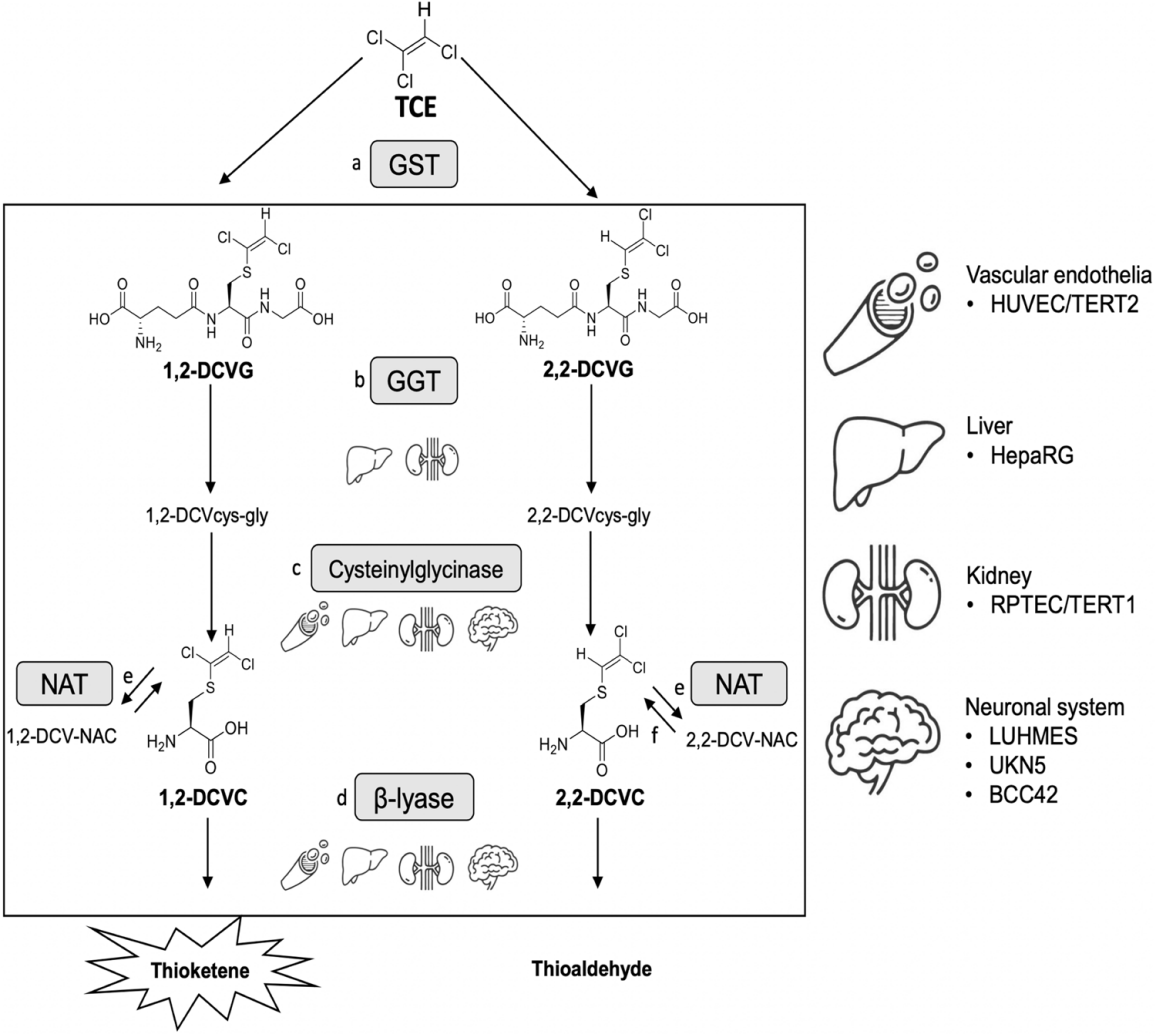
Diagram of mercapturic acid pathway of trichloroethylene (TCE) and human cell models tested. Enzymes involved: **a** glutathione S-transferases; **b** γ-glutamyltransferase; **c** cysteinyl-glycine dipeptidase; **d** cysteine conjugate β-lyase; **e** cysteine conjugate N-acetyltransferase; **f** aminoacylase. Formation of sulfoxides of cysteine conjugates and mercapturic acids is not shown to avoid an overcomplicated figure. The tested chemicals in this study were 1,2-DCVG, 2,2-DCVG, 1,2-DCVC and 2,2-DCVC. Human in vitro cell models tested: HUVEC/TERT2 as the endothelial vascular system, HepaRG as a hepatic model, RPTEC/TERT1 as a renal model, LUHMES as dopaminergic neurons model, UKN5 as hiPSC derived peripheral neurons and BCC42, as hiPSC derived brain cortical cultures containing all subtypes of neurons and astrocytes

For several chemicals the organ-specific toxic effects result from sequential metabolism in multiple tissues, strongly complicating mechanistic in vitro studies. An example of a chemical associated with toxic effects in multiple target organs is trichloroethylene (TCE), a synthetic halogenated hydrocarbon extensively used as an industrial solvent (Cichocki et al. 2016). Mechanistic in vivo and in vitro studies have shown that the hepatic and extrahepatic toxicities of TCE are caused by reactive metabolites and not by the parent compound itself (Lash et al. 2014). Biotransformation of TCE occurs mainly by oxidation by hepatic cytochrome P450s and, to lesser extent, via GSH-conjugation, also known as mercapturic acid pathway. GSH conjugation of TCE by human glutathione-S-transferases (GSTs) can lead to the formation of three regioisomeric GSH products (Capinha et al. 2021). Two regioisomers S-(1,2-*trans*-dichlorovinyl)-glutathione (1,2-DCVG) and S-(2,2-dichlorovinyl)-glutathione (2,2-DCVG) appeared to be the major regioisomers formed by human hepatic GSTs (Fig. 1). These conjugates can be further metabolized in the liver by biliary γ-glutamyltransferase (GGT) and/or transported to the rest of the body where they can be also metabolized by renal GGT. The resulting cysteinylglycine conjugates are subsequently converted to cysteine S-conjugates by dipeptidases (cysteinylglycinase) to S-(1,2-*trans*-dichlorovinyl)-L-cysteine (1,2-DCVC) and S-(2,2-dichlorovinyl)-L-cysteine (2,2-DCVC). For further bioactivation of DCVC, β-elimination by β-lyase has been described as the major bioactivation pathway resulting in the formation of ammonia, pyruvate and electrophilic species such as thioketenes and thioaldehydes (Fig. 1). Alternative pathways of DCVC bioactivation have been suggested, such as sulphoxidation by flavin-containing monooxygenases (FMO) (Cristofori et al. 2015).

The kidney is generally considered as the primary target organ for harmful effects of the GSH-conjugates of TCE (Lash et al. 2005; Lock and Reed 2006; Bruning and Bolt 2000). More recently, it has been proposed that GSH-derived conjugates of TCE may be involved in adverse effects of TCE in placenta (Elkin et al. 2018, 2019, 2021) and dopaminergic neurons (De Miranda et al. 2020). Additionally, 1,2-DCVG has been proposed to play a role in the neurotoxic effects of dichloroacetylene (Patel et al. 1993, 1994). Most of the mechanistic in vitro studies regarding the cytotoxicity of the S-conjugates of TCE have been focused on 1,2-DCVC and to lesser extent on its corresponding GSH-conjugate 1,2-DCVG. However, a recent study has demonstrated that 2,2-DCVG is the major GSH-conjugate formed in incubations of TCE with human liver fractions, formed at seven fold higher amounts than 1,2-DCVG (Capinha et al. 2021). In contrast to 1,2-DCVG, 2,2-DCVG has not yet been tested in in vitro studies. The only in vitro studies in which the toxicity of 1,2- and 2,2-regioisomers of TCE S-conjugates were compared involved DCVC and its corresponding mercapturic acid and were performed in isolated proximal tubular cells from rats (Commandeur et al. 1991) and mice (Newman et al. 2007), and rat perfused kidney (Ilinskaja and Vamvakas 1996). In these studies, these conjugates were incubated at relatively high concentrations (100, 500 and 2500 µM) and with only few cytotoxicity endpoints (LDH-leakage, methylglucose-uptake). An in vivo study using a single dose confirmed the higher toxicity of the 1,2-DCVC compared to 2,2-DCVC in rats, based on enzymuria and histopathology (Birner et al. 1997). So far, no comparative study of differences between 1,2- and 2,2-regioisomers has been performed using in vitro models of human origin.

Due to multi-site-organ metabolism, finding an ideal in vitro methodology for the comprehensive study of the mechanism of toxicity of metabolites from the GSH-conjugation pathway in different target organs is still challenging. In this study, we utilized six human in vitro cellular systems representing four different organs/tissues: kidney: RPTEC/TERT1 (Wieser et al. 2008); liver: HepaRG (Kanebratt and Andersson 2008); vascular endothelial system: HUVEC/TERT2 (Evercyte GmbH, Vienna, Austria); dopaminergic neuronal system: LUHMES cells (Scholz et al. 2011); peripheral neurons derived from human induced pluripotent stem cells (UKN5) (hiPSC) (Hoelting et al. 2016; Holzer et al. 2022), and 2D 42 days differentiated brain cortical cultures containing all subtypes of neurons and astrocytes (BCC42) (DB-ALM Protocol n° 207), (Fig. 1). The regioisomers of DCVG and DCVC were tested at a range of concentrations from 0.03 to 500 µM and with a 24 h exposure time. Our study compares for the first time the transcriptional stress responses from 1,2-DCVG, 2,2-DCVG, 1,2-DCVC and 2,2-DCVC across a panel of different human derived cell models, which will expand the mechanistic understanding of multi-organ toxicity resulting from GSH-conjugation of TCE. Additionally, this study provides evidence for the added value of in vitro model’s characterization and the suitability of high-throughput transcriptomics in predictive toxicology for chemicals that undergo bioactivation via the mercapturic acid pathway.

## Materials and methods

### Cell culture

HUVEC/TERT2, (Evercyte GmbH, Vienna, Austria) human umbilical vein endothelial cells were cultivated in EGM Endothelial Cell Growth Medium (Lonza CC-3124) supplemented with Endothelial Cell Growth Medium SingleQuots Supplements (CC-4133): endothelial growth supplement (EnG), hEGF, hydrocortisone, ascorbic acid and heparin plus 10% Fetal Bovine Serum (FBS). Cells were cultured in a controlled humidified 37 °C, 5% CO_2_ environment in plates coated with 0.1% gelatin solution (Sigma-Aldrich G1393) in phosphate-buffered saline solution (PBS). Cells were tested 1–2 days after reaching confluency in 96 well plates.

HepaRG cells were obtained from BioPredic International and were routinely cultured in William’s E GlutaMAX™ Supplement (Gibco 32,551,020) medium with additional 5 µg/ml insulin, 50 µM hydrocortisone 21-hemisuccinate, 100 U/ml penicillin, 100 µg/ml streptomycin and 9% FBS. Cells were cultured in a controlled humidified 37 °C, 5% CO_2_ environment and were differentiated according to BioPredic’s 6-day protocol (Biopredic 2017). Cells were tested 1 day after differentiation in culture medium not containing FBS.

RPTEC/TERT1 cells (Wieser et al. 2008; Aschauer et al. 2015) were routinely cultured in a 1:1 mix of DMEM (Gibco 11,966–025) and Ham’s F12 (Gibco 21,765–029) (containing a final concentration of 5 mM glucose) and supplemented with 2 mM Glutamax, 10 ng/ml epidermal growth factor, 36 ng/ml hydrocortisone, 5 μg/ml insulin, 5 μg/ml transferrin, 5 ng/ml selenium, 100 U/ml penicillin, 100 μg/ml streptomycin) (Jennings et al. 2009) and supplemented with a final concentration of 0.5% FBS. Cells were cultured and differentiated in a controlled humidified 37 °C, 5% CO_2_ environment, fed every 2/3 days and were tested at least 7 days after reaching confluency.

LUHMES (Lund human mesencephalic) cells were cultivated as described previously (Krug et al. 2013; Scholz et al. 2011; Schildknecht et al. 2013). In brief, cells were grown in standard cell culture flasks pre-coated with 50 µg/ml poly-L-ornithine and 1 µg/ml fibronectin (both from Merck, Darmstadt, Germany) in water. The maintenance culture was kept in proliferation medium consisting of advanced DMEM/F12 with 2 mM L-glutamine, 1 × N2-supplement, and 40 ng/ml FGF-2 (all from Thermo Fisher Scientific, Waltham, MA, USA). For differentiation, the medium was changed to differentiation medium consisting of advanced DMEM/F12 supplemented with 2 mM L-glutamine, 1 mM cAMP (Merck, Darmstadt, Germany), 1 µg/ml tetracycline (Merck, Darmstadt, Germany) and 2 ng/ml glia-derived neurotrophic factor (GDNF) (Bio-Techne, Minneapolis, MN, USA). The cells were kept at 37 °C with 5% CO_2_ until day 2 of differentiation. They were seeded on poly-L-ornithine- and fibronectin-coated 96-well plates at a density of 100,000 cells/cm^2^ and subsequently tested.

hiPSC derived peripheral neurons were differentiated from the Sigma iPSC0028 line (EPITHELIAL-1, #IPSC0028, Merck, Darmstadt, Germany) as described previously (Klima et al. 2021; Hoelting et al. 2016; Holzer et al. 2022). In brief, a combination of five small molecule pathway inhibitors (Noggin, SB-431642, CHIR99021, SU5402 and DAPT) was used to induce the differentiation to the sensory neuron phenotype. After 9 days of differentiation, immature peripheral neurons are cryopreserved in 90% FBS (Thermo Fisher Scientific, Waltham, MA, USA) and 10% dimethyl sulfoxide (DMSO; Merck, Darmstadt, Germany). Immature peripheral neurons were then thawed in culture medium composed of 75% N2-S medium (DMEM/F12, 1 × GlutaMax [both from Thermo Fisher Scientific, Waltham, MA, USA], 0.1 mg/ml apotransferrin, 1.55 mg/ml glucose, 25 μg/ml insulin, 20 nM progesterone, 100 μM putrescine and 30 nM selenium [all from Merck, Darmstadt, Germany]) and 25% KSR medium (knock out DMEM with 15% serum replacement, 1 × Glutamax, 1 × nonessential amino acids, and 50 µM β-mercaptoethanol [all from Thermo Fisher Scientific, Waltham, MA, USA]). 100′000 cells/cm^2^ were seeded on Matrigel-coated 96 well plates in culture medium supplemented with CHIR99021 (1.5 µM) (Axon Medchem, Groningen, Netherlands), SU5402 (5 µM) (Bio-Techne, Minneapolis, MN, USA) and DAPT (5 µM) (Merck, Darmstadt, Germany) and tested between day 0 and 1 of differentiation.

hiPSC-derived cortical neuronal-glia culture (BCC42) were differentiated from the SBAD2 hiPSCs obtained during the IMI-funded StemBANCC project (http://stembancc.org) as previously described (Morrison et al. 2015). hiPSCs were differentiated toward neuronal stem cells using the dual SMAD inhibition protocol (Chambers et al. 2009). In brief, NSCs were isolated from the rosettes and maintained and propagated appropriately. The neural progenitor cells (NPCs) were cryopreserved after 6–8 passages, when it has reached the appropriate cell number, and characteristics (Zhou et al. 2016). For more details see DB-ALM Protocol No. 214 and No. 215. Terminal differentiation of neuronal culture consisted of NPCs seeding on Poly-L-Ornithine/Laminin (POL/L) (Merck) coated dishes and maintained until reaching confluency. Confluent cultures were detached by adding Accutase solution (Merck), re-suspended in neural maintenance medium (NMM) and plated on POL/L-coated clear bottom 96-well assay plates. The medium was replaced with fresh NMM every other day. Antioxidants-free neural maintenance medium (NMM-AO) was used after TD30. The cells were already characterized previously, but briefly, the culture contains matured astrocytes and different neuronal subtypes after 42-days of terminal differentiation, it possesses characteristic of a more matured, active neuronal co-culture (Kobolak et al. 2020; Snijders et al. 2021). Cell cultures were differentiated for 42 days before the chemical exposure.

### Chemicals

The synthesis, purification and characterization of the used regioisomers of GSH S-conjugates (1,2-DCVG, 2,2-DCVG) and cysteine S-conjugates (1,2-DCVC, 2,2-DCVC) are described in detail in our recent study (Capinha et al. 2021). Each laboratory involved in this study received crystals from the same batches of the four regioisomers (purity > 98% based on HPLC-analysis). A 1 mM stock solution of each TCE-conjugate was prepared and diluted in the complete cell culture medium from each corresponding cell system. CDDO-Me (Cayman chemicals, 11,883) was included as positive control for oxidative stress induction in the TempO-Seq assay and dissolved in dimethyl-sulfoxide (DMSO). CDDO-Me final treatment solution 0.1% (v/v) DMSO for all tested models. Media untreated samples were used as controls of all treated samples including CDDO-Me. Chemical exposures were performed with either freshly made solutions or thawed once after storage at  – 20 °C. All purchased chemicals were obtained from Sigma-Aldrich (St. Louis, MO, USA.) if not stated otherwise.

### Viability assays

Cell viability upon 24 h exposure of seven to eleven concentrations (0, 0.03, 0.09, 0.29, 0.9, 2.9, 9.3, 29.7, 62.5, 125, 250 and 500 µM) of each chemical was evaluated by different assays and shown as percentage of medium control. Concentration–response curve fitting (*exponential—one phase decay*) and data analysis was performed using Prism software (GraphPad 9.0). Grey background in the viability assay plots depicts ≥ 25% cytotoxicity.

#### Resazurin reduction assay

Viability of HUVEC/TERT2, HepaRG and RPTEC/TERT1 cells were determined using the resazurin reduction assay, as described previously (Jennings et al. 2007; van der Stel et al. 2020). In summary, after 24 h exposure to DCVGs and DCVCs in a 96-well format, cell supernatant was replaced with 100 µl/well of resazurin medium solution of 44 µM concentration for 1 h at 37 °C and 5% CO_2_. The fluorescent product from resazurin reduction, resorufin, was detected at 540/590 nm excitation/emission using a CLARIOstar plate reader.

#### LUHMES and UKN5 assays

The test compounds were added to the cells 1 h after seeding for an exposure time of 24 h. The cells were stained with 1 µg/ml Hoechst (H-33342) and 1 µM calcein-AM 1 h before readout. Imaging was performed automatically using an ArrayScan VTI HCS microscope (Thermo Fisher Scientific, Waltham, MA, USA). Images were analyzed for neurite area and cell viability by an automated algorithm exactly as described previously (Hoelting et al. 2016; Stiegler et al. 2011). In brief, H-33342 staining was used to identify individual cell nuclei. The somatic area was defined by enlarging the nuclear area, and subtracted from the calcein stain. The remaining calcein-positive pixels corresponded to the neurite area. Cell viability was assessed using the same images. Cells that were double-positive for H-33342 and calcein were classified viable, cells only positive for H-33342 as dead.

#### ATP assay

ATP viability assay was performed with the CellTiter-Glo® Luminescent Cell Viability Assay Kit (Promega) according to the manufacturer’s description. The luminescent signal was recorded with a Thermo VarioScan Flash Multimode Plate Reader (Thermo Fisher Scientific).

#### Transcriptomics with TempO-Seq assay

Cells were cultured as described above and exposures to TCE conjugates were performed in 96-well plate format. All conditions were tested in triplicates. The concentrations selected of each chemical for treatment in each cell type correspond to the highest of one to seven concentrations (up to 500 µM) with  ≤ 25% decrease in viability, upon 24 h exposure. After exposure to TCE conjugates, cells were washed once with PBS (Gibco 14,190–094) and lysed subsequently with TempO-Seq lysis buffer (BioClavis, Glasgow, Scotland) for 15 min at room temperature. All samples were immediately transferred and stored at  – 80 °C until shipped to BioClavis (Glasgow, Scotland) for TempO-Seq analysis. A panel of ≈ 3500 genes, denominated EUToxRisk v2.2 was used as a surrogate for the whole transcriptome and is considered sufficient to cover well-characterised biological pathways for toxicological studies.

BioClavis returned the data as a data matrix of gene expression in raw counts per probe per sample, after internal quality control analysis as described elsewhere (Limonciel et al. 2018).

### Sample quality check and statistical analysis of TempO-Seq data

The raw read counts' sample quality check was performed where samples with a total count across gene probes below 200,000 were considered poorly sequenced and therefore removed from the dataset. In addition, Pearson’s correlation coefficient between replicates were calculated with the corr function from the Pandas package (*Python*) to evaluate intragroup sample variability. Pearson’s correlation coefficient ≤ 0.75 within replicates were considered outliers and were removed from the dataset. The resulting data matrix was utilized for different types of transcriptional analysis, see Figure S1 (electronic Supplementary file 1). Media control samples were utilized for a principal component analysis (PCA) performed after rlogtransformation (regularized log transformation, a *DES*eq2 function, for normalisation with respect to library size) of the raw read counts.

Additionally, specific cell markers/enzymes potentially relevant for TCE biotransformation were investigated across cell models from the mRNA raw counts. Differential expression analysis (pair-wise comparison, condition vs medium control) per cell type were acquired through *DESeq2 Bioconductor* package (Online Resources 2—Table S1) (Love et al. 2014; Nunes et al. 2022). For differently expressed genes (DEGs) cut-off, the following filters were applied: padj < 0.01, basemean > 35 and log2 fold change >| 0.58 |. Upregulated genes (padj > 0.01, basemean > 35 and log2 fold change > 0.58) *versus* background gene panel list (EUToxRisk v2.2) was used for over-representation analysis with the ConsensusPathDB (CPDB) online tool (http://cpdb.molgen.mpg.de) with a minimum overlap of 2 probes and p-value cut-off of 0.01, as previously described (Nguyen et al. 2021; Nunes et al. 2022). Over-representation analysis was performed for two conditions: *low concentrations* and *high concentrations* of 1,2-DCVC tested. The lowest concentrations tested demonstrating at least one gene upregulated in each model were considered for *low concentrations* assessment. The upregulated genes from the highest concentrations tested of 1,2-DCVC, per cell model, were combined and used as input for over-representation analysis for *high concentrations* assessment. For each type of exposure, the upregulated DEGs (padj < 0.01, basemean > 35 and log2FC > 0.58) from each cell type were combined and repeated genes were removed. The CPDB platform integrates different relevant databases of signalling, metabolic pathways and protein interactions such as *Wikipathways, Smpdb, Kegg, Reactome, Pharmagkb, Pid, Biocarta, Ehmn, Humancyc, Inoh, Netpath* and *Signalink.* Each database may contain different ID pathways for the same type of cellular response. Thus, to facilitate the visualisation of different types of cellular responses, redundant pathways were removed, and the ones with lowest q-value are displayed. Gene representation of specific stress response pathways was performed with the mRNA raw counts from the exposure to the four TCE conjugates to evaluate the potential concentration-dependent responses across cell models of selected genes found during over-representation  analysis. Concentration–response data for selected DEGs and mRNA raw counts were plotted in Prism software (GraphPad 9.0). The remaining analysis and figures were made using  the following *R* packages: * dplyr, reshape, ggplot2, UpSetR and ggpubr.*

## Results

### Assessment of basal transcriptomic differences among cell models

**Fig. 2:**
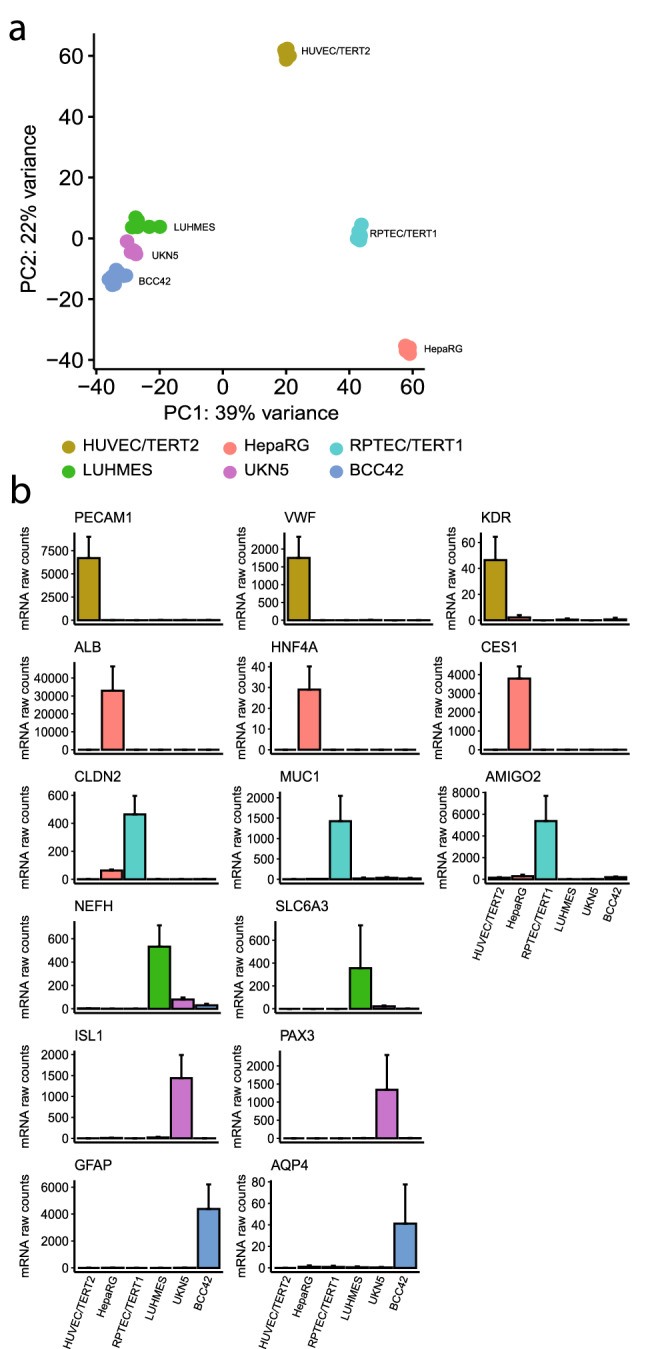
Basal transcriptomic differences across cell models **a** Principal Component Analysis (PCA) using rlog transformed expression values of mRNA counts. Untreated samples (medium controls) from each cell system were used as input for PCA. Each dot represented a sample, color-coded by cell type: HUVEC/TERT2, HepaRG, RPTEC/TERT1, LUHMES, UKN5, BCC42. **b** mRNA baseline expression of genes representative of each cell model from untreated samples: CD31 protein-coding gene (PECAM1), Von Willebrand factor (VWF), kinase domain receptor (KDR), albumin (ALB), hepatocyte nuclear factor 4 alpha (HNF4A), carboxylesterase 1 (CES1), claudin 2 (CLDN2), mucin 1 (MUC1), Adhesion Molecule With Ig Like Domain 2 (AMIGO2), neurofilament heave polypeptide gene (NEFH), solute carrier family 6, A3 (SLC6A3), insulin gene enhancer protein (ISL1), paired-box homeotic gene 3 (PAX3), aquaporin-4 (AQP4), and glial fibrillary acidic protein (GFAP)

In order to have an overview of the high-dimensional differences in the transcriptomic signature of the cell systems tested, an unsupervised PCA was performed on the media control samples converted by regularized log transformation (rlogtransformed) of each cell model that passed the quality control workflow. The PCA resulted in a clear separation of the four organs represented by the six cell models. HUVEC/TERT2, HepaRG and RPTEC/TERT1, as endothelial, hepatic, and renal models, respectively, were separated from each other (Fig. 2a). The three neuronal models, LUHMES, UKN5 and BCC42, clustered closely, indicating similarities among their transcriptomic profile.

Figure 2b shows the mRNA raw counts of media control samples that were used to compare the basal expression of selected genes which were shown to be specifically expressed in the six cell models used in the current study. The renal proximal tubule model RPTEC/TERT1 showed a high expression of genes encoding claudin 2 (CLDN2), mucin 1 (MUC1) and Adhesion Molecule With Ig Like Domain 2 (AMIGO2), as previously demonstrated (Limonciel et al. 2018). Differentiated HepaRG, an hepatocyte-like cell model, showed a high expression of genes encoding hepatic proteins such as albumin (ALB), hepatocyte nuclear factor 4 alpha (HNF4A) and xenobiotic metabolising enzymes, such as carboxylesterase 1 (CES1). In HUVEC/TERT2, endothelial specific encoding genes that were found to be highly expressed, compared with the other cell types, were CD31 protein coding gene (PECAM1), Von Willebrand factor (VWF), and the kinase domain receptor (KDR), which functions as the main mediator of VEGF-induced endothelial proliferation (Müller et al. 2002; Kroll and Waltenberger 1997) (Fig. 2b). LUHMES cells showed a specific expression of the genes of neurofilament heavy chain (NEFH) and the transporter SLC6A3, belonging to the solute carrier family 6, which is widely distributed throughout the brain in areas of dopaminergic activity (A. K. Krug et al. 2014). The insulin gene enhancer protein (ISL1) and paired-box homeotic gene 3 (PAX3), which has been documented as a peripheral neuron subtype specifier and found in developing peripheral nervous system, respectively, were found to be exclusively expressed in the UKN5 model. Finally, the BBC21 cell culture showed a high expression of aquaporin-4 (AQP4) and the glial fibrillary acidic protein (GFAP) demonstrating the presence of matured astrocytes in the culture (Zhou et al. 2016).

### Effects on viability from exposure to DCVG and DCVC regioisomers

**Fig. 3:**
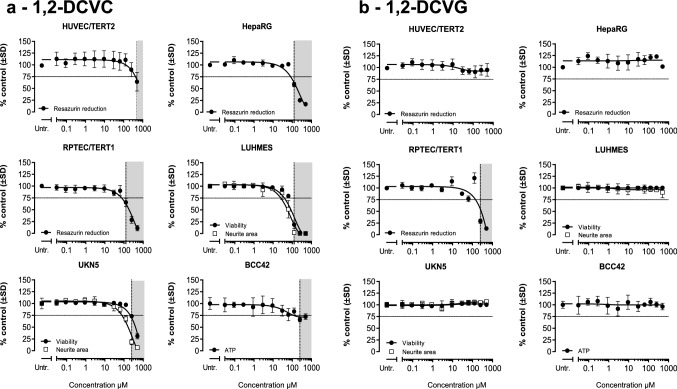

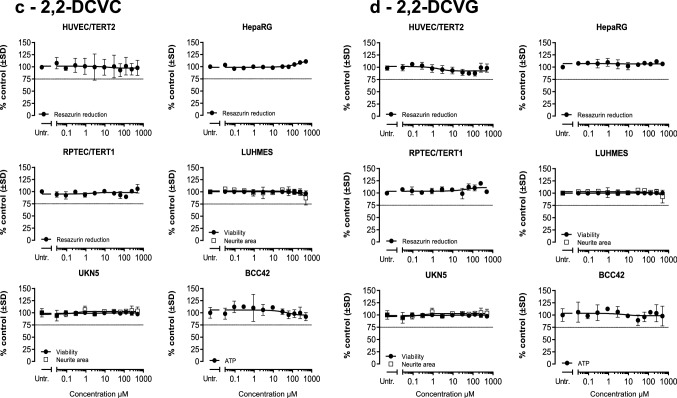
Cell viability assessment after DCVGs and DCVCs exposure. Measurements were performed after 24 h exposure of eleven concentrations (0.03, 0.09, 0.29, 0.9, 2.9, 9.3, 29.7, 62.5, 125, 250, 500) of indicated TCE conjugates in the six human cell models tested. Viability in HUVEC/TERT2, HepaRG and RPTEC/TERT1 was assessed by resazurin reduction. Viability and neurite area in LUHMES and UKN5 were assessed with Calcein-AM and H-33342 staining. ATP content was used as viability parameter in BCC42 cells. Values represent the mean % medium control ± SD for each different assay. Untr: untreated samples were used as negative controls. **a** 1,2-DCVC, **b** 1,2-DCVG, **c** 2,2-DCVC, **d** 2,2-DCVG

To evaluate the sensitivity of the six cell models towards the two regioisomers of DCVG and DCVC, viability assays were performed after exposure for 24 h to a wide range of concentrations (up to 500 µM) of each compound. Concentrations resulting in more than 25% decrease in viability were considered cytotoxic and are depicted with grey background in the viability plots (Fig. 3). The lowest concentrations leading to more than 25% loss of viability were designated as point of departure (POD).

1,2-DCVC was found to decrease viability in all tested systems with RPTEC/TERT1, HepaRG and LUHMES as the most sensitive models with the lowest POD of 62.5 µM (Fig. 3a). The remaining models exposed to 1,2-DCVC displayed a POD of 125 µM, except for HUVEC/TERT2, with a POD of 500 µM. In contrast, 2,2-DCVC did not cause a significant decrease in viability in any cell model, even after exposure to 500 µM (Fig. 3c).

1,2-DCVG affected the viability of RPTEC/TERT1 to the same extent as 1,2-DCVC, whereas the other cell models show no significant decrease in viability (Fig. 3b). Just like 2,2-DCVC, its corresponding GSH-conjugate 2,2-DCVG did not cause a significant decrease in cell viability for any of the cell types at any of the tested conditions (Fig. 3d).

### TempO-Seq analysis of differentially expressed genes in different cell models

**Fig. 4:**
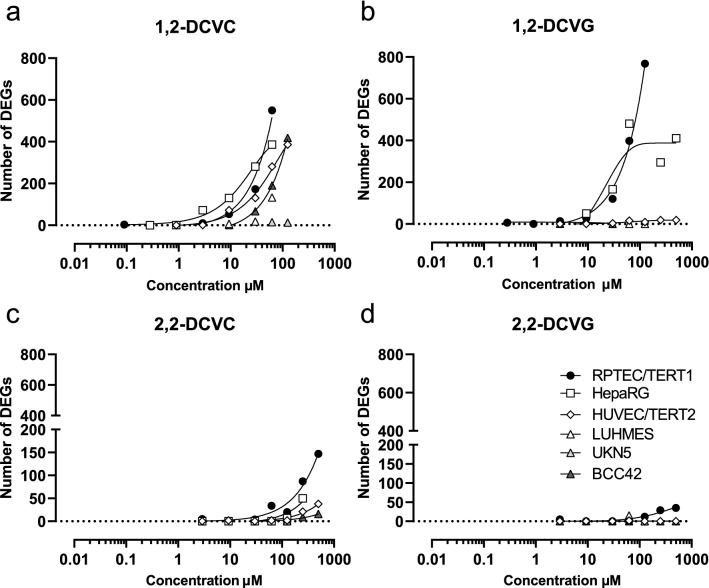
Number of differentially expressed genes (DEGs) for tested DCVG and DCVC regioisomers in the six human cell models for a 24 h exposure. Number of DEGs consists of number of genes, up and down regulated, (padj < 0.01, basemean > 35 and log2FC >| 0.58 |) after DESeq2 package analysis in *R* for each condition vs corresponding untreated control

Due to the differences in effects on cell viability, the TempO-Seq analysis of differentially expressed genes (DEGs) were only applied on concentration of S-conjugates in which the cell viability was still higher than 75%. These conditions facilitate the investigation of early cellular transcriptomic responses and minimize unspecific gene changes due to cell death. Analysis of DEGs by quantification of mRNA alterations, relative to media-control samples, plays a key role in understanding the molecular basis of phenotypic variation between different conditions. Figure 4 shows the concentration dependent increase of the number of DEGs caused by the four S-conjugates in the six cell models.

As shown in Fig. 4a, exposure to 1,2-DCVC cause a concentration-dependent increase in number of DEGs in all cell models. At 62.5 µM, a concentration tested for all cell models, RPTEC/TERT1 and HepaRG were found to have the highest number of DEGs, 550 and 386 respectively, followed by BCC42 (191), LUHMES (133), HUVEC/TERT2 (44) and UKN5 (15).

After 1,2-DCVG exposure, RPTEC/TERT1 and HepaRG were the most affected models with 768 and 411 DEGs, respectively, at their top concentrations, Fig. 4b. The other cells models were less sensitive to 1,2-DCVG with 19 DEGs for HUVEC/TERT2, 6 for BCC42 and no DEGs found for LUHMES. UKN5 was not tested for 1,2-DCVG because it was unresponsive to 1,2-DCVC.

As shown in Fig. 4c, the exposure of cells to 2,2-DCVC led to a much lower number of DEGs in RPTEC/TERT1, HepaRG and HUVEC/TERT2, compared to the 1,2-DCVC regioisomer. At 500 µM, 147 DEGs were found for RPTEC/TERT1 and 16 for BCC42. At 125 µM, 7 DEGs were found for HepaRG, 7 for LUHMES. No DEGs were found in UKN5.

RPTEC/TERT1 was the only cell model responsive in a concentration dependent manner to 2,2-DCVG and showed a lower number of DEGs compared to 2,2-DCVC, with 12, 29 and 35 DEGs at 125, 250 and 500 µM respectively, Fig. 4d.

### Over-representation analysis by ConsensusPathDB-human of 1,2-DCVC exposure

An over-representation analysis using the ConsensusPathDB-human database (CPDB) (http://cpdb.molgen.mpg.de) (Kamburov et al. 2011) was performed to investigate the stress response pathways over-represented due to 1,2-DCVC exposure. The analysis was performed for two levels of exposures: *low concentration,* as lowest concentrations of 1,2-DCVC tested across cell models with at least one upregulated DEG, and *high concentration*, as highest concentrations of 1,2-DCVC tested. At *low concentrations* of 1,2-DCVC, the most over-represented pathway found was the *NRF2 pathway* from Wikipathways (Table 1) with 10.4% of upregulated genes overlapping with the total NRF2 gene set, including Heme Oxygenase 1 (HMOX1), NAD(P)H Quinone Dehydrogenase 1 (NQO1), Gamma-Glutamylcysteine Synthetase Regulatory Subunit (GCLM) and solute carrier family 7 member 11 (SLC7A11). *Nuclear receptors meta-Pathway* and other pathways related to cellular and xenobiotic metabolism (e.g., *Glucuronidation*, *Phase II—conjugation of compounds, Drug metabolism – other enzymes),* were also found at *low concentrations* of 1,2-DCVC exposure. Regarding the exposure of *high concentrations* of 1,2-DCVC, the most over-represented pathway was also the *NRF2 pathway* with an increase from 10.4% to 39% genes overlap with the pathway gene set list, followed by *Photodynamic therapy-induced unfolded protein response (66.7% genes) and EIF2AK1 (HRI) to heme deficiency (81.8% genes)*. Other stress response pathways were found, such as, *IL-17 signalling pathways – (human)* for inflammation and *p53 signalling pathway – (human)*. For the complete lists of stress response pathways at low and high concentrations, see electronic Supplementary file 2.

**Table 1:** Over-representation analysis of 1,2-DCVC exposure across cell models for low and high concentrations

Low concentrations			
q-value	% genes overlap*	Pathway	Pathway source
1.96E-07	10.4	NRF2 pathway	Wikipathways
2.25E-05	4.8	Nuclear Receptors Meta-Pathway	Wikipathways
5.89E-05	18.2	Porphyrin and chlorophyll metabolism—(human)	KEGG
0.000425441	23.1	Glucuronidation	Reactome
0.000472089	21.4	Codeine and Morphine Metabolism	Wikipathways
0.000576877	18.8	Pentose and glucuronate interconversions—(human)	KEGG
0.000658341	8.4	Phase II—Conjugation of compounds	Reactome
0.000658341	16.7	Ascorbate and aldarate metabolism—(human)	KEGG
0.000658341	15.8	Ferroptosis—(human)	KEGG
0.004668623	7.5	Drug metabolism—other enzymes—(human)	KEGG

High concentrations			
q-value	% genes overlap*	Pathway	Pathway source
3.90E-05	39	NRF2 pathway	Wikipathways
0.00019879	66.7	Photodynamic therapy-induced unfolded protein response	Wikipathways
0.000285382	81.8	Response of EIF2AK1 (HRI) to heme deficiency	Reactome
0.000285382	25.4	Cellular responses to stress	Reactome
0.000500637	39.6	Protein processing in endoplasmic reticulum—(human)	KEGG
0.001395995	57.9	Ferroptosis—Homo sapiens (human)	KEGG
0.002145114	37	IL-17 signaling pathway—(human)	KEGG
0.004475598	100	Cysteine Metabolism	SMPDB
0.005007318	36	p53 signaling pathway—(human)	KEGG
0.015686101	44	Validated transcriptional targets of TAp63 isoforms	PID

Examples of 10 significant (q value ≤ 0.01) pathways from over-representation analysis among cell models after chemical exposure to 1,2-DCVC for 24 h. Upregulated DEGs across cell models upon 1,2-DCVC exposure were used as input for ORA statistical analysis. Low concentrations were considered the lowest concentration tested that resulted in at least one DEG for each cell model. High concentrations were considered as the top concentration tested in each cell model. *% genes overlap— % of DEGs in tested condition that overlap with pathway gene set list that are contained in the background list of genes (EUToxRisk v2.2)

### Intersection of upregulates genes across models for 1,2-DCVC and 1,2-DCVG exposure

**Fig. 5:**
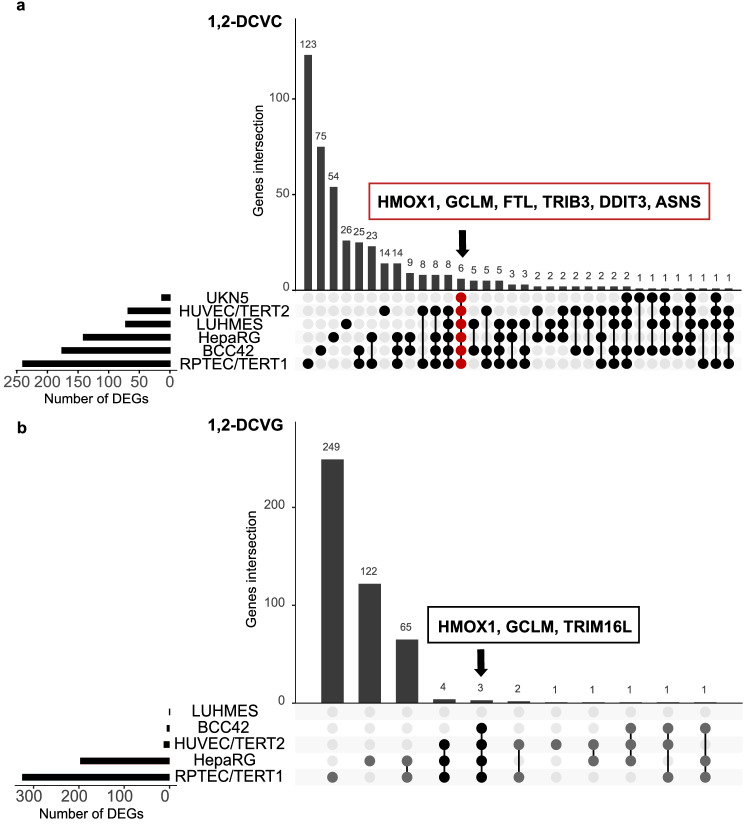
Intersection of upregulated DEGs for the top concentrations of 1,2-DCVC and 1,2-DCVG exposure across cell models. Number of DEGs: number of upregulated DEGs found for cell model (padj < 0.01, basemean > 35, log2FC > 0.58). Genes intersection: Number of DEGs found in common for depicted cell models (black or red dots) **a** 1,2-DCVC exposure. Six genes found commonly upregulated across the cell panel: Heme Oxygenase 1 (HMOX1), Glutamate-Cysteine Ligase (GCLM) and Ferritin Light Chain (FTL), DNA Damage Inducible Transcript 3 (DDIT3), Tribbles Pseudokinase 3 (TRIB3) and Asparagine Synthetase (Glutamine-Hydrolyzing) (ASNS). **b** 1,2-DCVG exposure. UKN5 were not tested for 1,2-DCVG exposure and therefore not included in the analysis. No DEGs were found after 1,2-DCVG exposure in LUHMES. RPTEC/TERT1, HUVEC/TERT2 and HepaRG demonstrated three genes commonly upregulated, HMOX1, GCLM and Tripartite Motif Containing 16 Like (TRIM16L)

The differences and similarities of DEGs resulting from exposures of 1,2-DCVC and 1,2-DCVG per cell model are presented in Fig. 5. After 1,2-DCVC exposure, the cell models with the highest number of common upregulated DEGs were RPTEC/TERT1 and BCC42 with 25 DEGs in common, followed by 23 DEGs intersecting in RPTEC/TERT1 and HepaRG. Among the six cell models, 6 genes were found to be commonly differentially up regulated, namely HMOX1, GCLM, FTL, Tribbles Pseudokinase 3 (TRIB3), DNA Damage Inducible Transcript 3 (DDIT3) and Glutamine-Dependent Asparagine Synthetase (ASNS) (Fig. 5a).

Upon exposure to 1,2-DCVG, RPTEC/TERT1 and HepaRG shared the highest number of common DEGs with 65 genes. No DEGs were found after 1,2-DCVG exposure in LUHMES cells. In the responsive models to 1,2-DCVG, HepaRG, RPTEC/TERT1, HUVEC/TERT2 and BCC42, the intersection of three genes was found, namely HMOX1, GCLM and TRIM16L (Fig. 5b).

### Concentration dependent effects of S-conjugates on raw mRNA counts of DEGs

Based on the over-representation  statistical analysis from 1,2-DCVC exposure (Table 1), three stress response pathways were selected for further investigation: *Nrf2* response, UPR response and P53 pathway related response. First, mRNA counts of three genes representative for each pathways were selected to investigate the concentration-dependent response after 24 h exposure to 1,2-DCVC, 1,2-DCVG, 2,2-DCVG and 2,2-DCVC. HMOX1, GCLM and FTL were selected for *Nrf2* pathway representation, TRIB3, DDIT3 and ASNS as UPR representatives and Growth Arrest and P53 pathway Inducible alpha (GADD45A), E3 Ubiquitin-Protein Ligase MDM2 (MDM2) and Snail Family Transcriptional Repressor 2 (SNAI2), representing DNA damage related response.

#### Nrf2-response

**Fig. 6:**
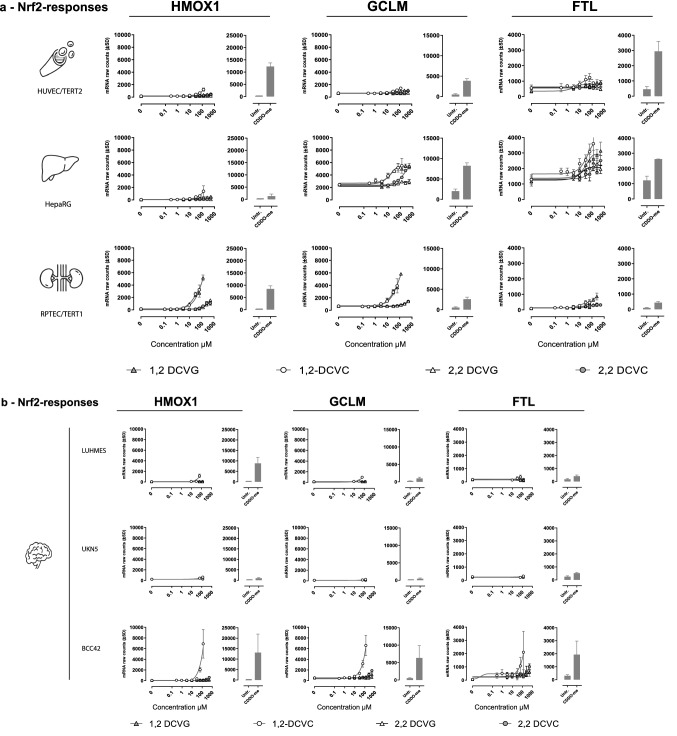

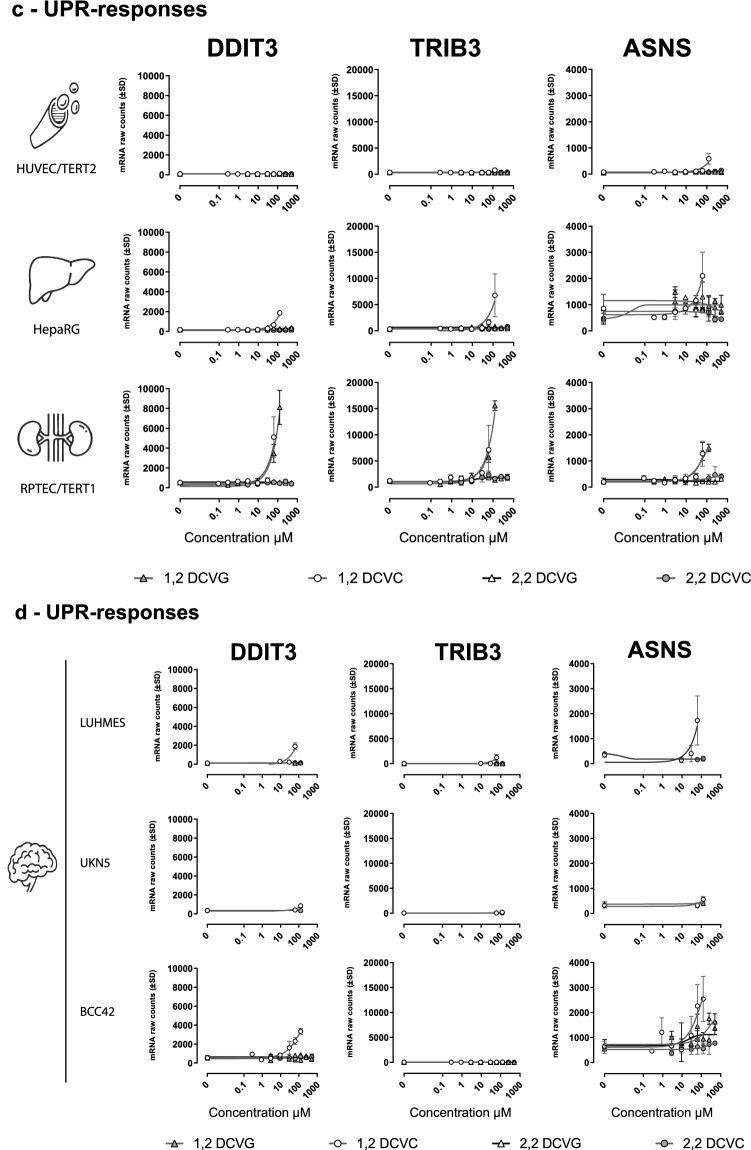

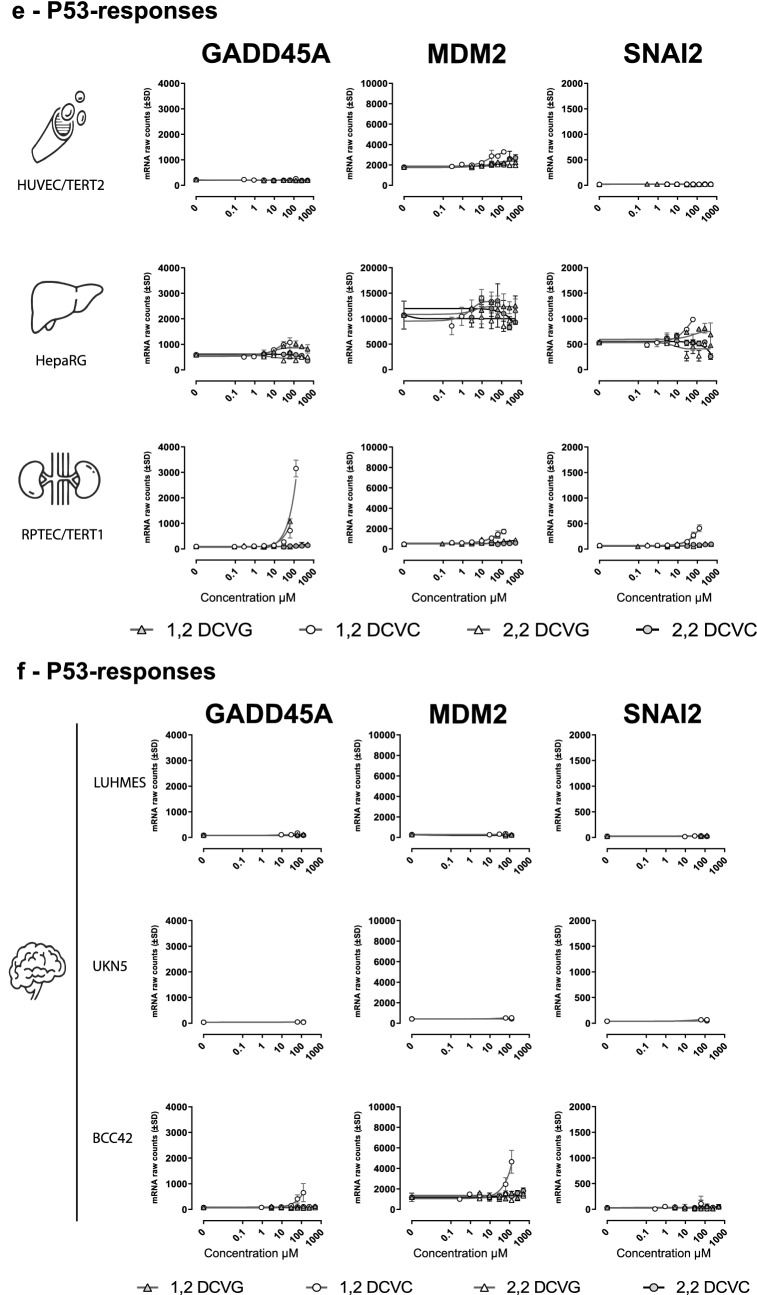
TCE conjugates concentration vs mRNA raw counts response of selected gene representing three major stress response pathways suggested by over-representation analysis using CPDB. **a** Nrf2—Oxidative stress: Heme Oxygenase 1 (HMOX1), Glutamate-Cysteine Ligase (GCLM) and Ferritin Light Chain (FTL). **b** UPR- Unfolded protein response: Inducible Transcript 3 (DDIT3), Tribbles Pseudokinase 3 (TRIB3) and Asparagine Synthetase (Glutamine-Hydrolyzing) (ASNS). **c** P53-responses: Growth Arrest And DNA Damage Inducible Alpha (GADD45A), MDM2 Proto-Oncogene (MDM2), Snail Family Transcriptional Repressor 2 (SNAI2)

The six tested models showed a concentration dependent Nrf2-response after 1,2-DCVC exposure although to different extents, as visualized in Fig. 6a. The RPTEC/TERT1 cells showed the highest Nrf2-response after exposure to 1,2-DCVC and 1,2-DCVG, with significant increase of mRNA-levels of HMOX1, GCLM and to lesser extent FTL, Fig. 6a. In RPTEC/TERT1 cells, 1,2-DCVC and 1,2-DCVG showed almost identical concentration-dependency and comparable levels of increase of mRNA counts of these three DEGs. When compared to RPTEC/TERT1 cells, only a small increases in Nrf2-response were observed in HepaRG and HUVEC/TERT2 cells after exposure to 1,2-DCVC. In these cells, 1,2-DCVG also showed effects although at higher concentration than in RPTEC/TERT1.

The 2,2-regioisomers of DCVC and DCVG showed much smaller Nrf2-responses than their corresponding 1,2-regioisomers. Since the 2,2 regioisomers did not decrease cell viability, Fig. 3, cellular responses could be analyzed up to 500 µM concentration. At the highest concentration the 2,2-regioisomers produced a significant Nrf2-response, although still to a much lower extent than that observed at 30 µM of the 1,2-regioisomers.

Of the three neuronal cell models, BCC42 showed the highest increase in raw mRNA counts of HMOX1, GCLM and FTL when exposed to 1,2-DCVC. At 30 µM 1,2-DCVC the response was comparable to that observed with 10 µM in RPTEC/TERT1 cells, Fig. 6a. In contrast to RPTEC/TERT1 cells, exposure to 1,2-DCVG, showed only a very small increase in these Nrf2-responses at the highest concentrations (250 and 500 µM) tested in BCC42 cells. Of the 2,2-regioisomers only 2,2-DCVC showed a relatively weak Nrf2-response at the highest concentration. Its GSH-conjugates, 2,2-DCVG did not show any effect.

LUHMES cells only showed a low increase in mRNA of Nrf2-associated DEGs at the highest concentrations of 1,2-DCVC tested. The corresponding GSH-conjugate, 1,2-DCVG, and the 2,2-regioisomers 2,2-DCVC and 2,2-DCVG did not show an increase in mRNA-counts.

UKN5 cells, which was only exposed to 62,5 and 125 µM of 1,2-DCVC, appeared to be the least sensitive neuronal cell model for *Nrf2* response. 1,2-DCVG, 2,2- DCVC and 2,2-DCVG showed no effects in this cell model.

#### UPR-response

As shown in Fig. 6b, both 1,2-DCVC and 1,2-DCVG showed comparable concentration-dependent increases in mRNA counts of DDIT3, TRIB3, and ASNS in RPTEC/TERT1 cells. The HepaRG cells also showed an increase in these mRNA levels, although to a much lower extent compared to the RPTEC/TERT1 cells. The HUVEC/TERT2 cells were the least sensitive of the non-neuronal cells, showing only an increase of mRNA of ASNS at the highest concentration of 1,2-DCVC. In the neuronal models, only 1,2-DCVC was shown to produce an UPR-response in LUHMES and BCC2 cells, Fig. 6b. UKN5-cells showed no UPR-response to 1,2-DCVC and 1,2-DCVG at the two concentrations tested.

The 2,2-regioisomers of DCVC and DCVG did not show an UPR-response in any of the cell models tested.

#### DNA-damage response

For the P53 pathway related responses, RPTEC/TERT1 and BCC42 appeared the most affected models. At the highest concentrations of 1,2-DCVC used, 62.5 µM and 125 µM, respectively, a significant increase in mRNA levels of both GADD45A, MDM2 and SNAI2 was observed, Fig. 6c. In the neuronal BCC42-cells only increases of GADD45A and MDM2 expression was observed, Fig. 6f.

The 2,2-regioisomers of DCVC and DCVG did not produce a response of the genes involved in the p53 signalling pathway in any of the cell models studied.

### Concentration dependent effects of S-conjugates on fold increases of DEGs

The basal mRNA levels of the stress response genes appeared to vary strongly between cell models. We therefore also analyzed the fold increases of DEGs, by comparing mRNA levels of DEGs in compound-treated cells with the corresponding mRNA levels in media control cells, which were treated with cell medium only. Table 2 shows the fold increases of DEGs in cells exposed to 62,5 µM of 1,2-DCVC, a non-toxic concentration used in all six cell models. Next to the 9 DEGs presented in Fig. 6, a selection of 17 additional DEGs were included that also show large fold increases in the six cell models.

**Table 2:** Comparison of fold increases of a selection of differentially expressed genes in cell models exposed for 24 h to 62,5 µM 1,2-DCVC

Differentially expressed genes	Associated stress response pathway(s)	RPTEC/ TERT1	HepaRG	HUVEC/ TERT2	LUHMES	BCC42	UKN5
*HMOX1*	*a, d*	23	13	3.9	22	**35**	2.0
*GCLM*	*a, d*	6.0	2.4	1.9	**8.1**	6.1	< 1.5
*FTL*	*a, d*	**4.6**	2.5	1.7	4.1	3.9	< 1.5
*UGT1A6*	*a, e*	**8.7**	< 1.5	< 1.5	< 1.5	< 1.5	< 1.5
*UGT1A8*	*a*	**7.8**	< 1.5	< 1.5	< 1.5	< 1.5	< 1.5
*UGT1A10*	*a*	**7.9**	< 1.5	< 1.5	< 1.5	< 1.5	< 1.5
*SRXN1*	*a*	7.0	4.6	1.7	< 1.5	**21**	< 1.5
*CEBPB*	*a*	3.0	< 1.5	1.6	**112**	4.2	< 1.5
*SLC7A11*	*a*	< 1.5	**7.7**	2.7	< 1.5	5.6	< 1.5
*NMRAL2P*	*a*	25	< 1.5	9.2	< 1.5	**33**	< 1.5
*OSGIN1*	*a*	14	3.9	2.3	< 1.5	**30**	< 1.5
*NQO1*	*a*	8.4	< 1.5	2.6	**15**	4.4	2.3
*TXNRD1*	*a, e*	**21**	< 1.5	1.9	3.0	4.3	< 1.5
*DDIT3*	*b*	7.7	4.4	< 1.5	**17**	5.8	< 1.5
*ASNS*	*b*	5.3	3.1	< 1.5	**7.8**	4.0	< 1.5
*TRIB3*	*b*	4.5	5.3	< 1.5	**70**	10	< 1.5
*TRIM16L*	*b*	6.6	1.8	3.8	6.3	**8.1**	< 1.5
*GDF15*	*b, g*	7.0	5.7	< 1.5	11	**16**	< 1.5
*GADD45A*	*c*	**11**	1.8	< 1.5	< 1.5	5.7	< 1.5
*SNAI2*	*c*	**4.3**	2.1	< 1.5	< 1.5	< 1.5	< 1.5
*MDM2*	*c*	< 1.5	< 1.5	1.6	< 1.5	**5.1**	< 1.5
*TNFRSF10B*	*c*	1.9	< 1.5	< 1.5	**4.1**	3.0	< 1.5
*SLC7A5*	*e*	4.7	5.1	5.5	**40**	< 1.5	< 1.5
*ME1*	*e*	1.8	< 1.5	< 1.5	**13**	< 1.5	< 1.5
*CHAC1*	*f*	< 1.5	< 1.5	< 1.5	< 1.5	**45**	< 1.5
*ATF5*	*f*	< 1.5	2.1	< 1.5	**29**	< 1.5	< 1.5

Numbers represent fold-increases of DEGs relative to the medium controls; numbers in bold represent the largest fold increase across the six cell models; associated stress pathways correspond to pathways found from over-representation analysis using the online tool http://cpdb.molgen.mpg.de from 1,2-DCVC exposures; a, Nrf2-response; b, UPR-response; c, p53-signalling; d, Ferroptosis; e, Nuclear receptor Meta-pathway; f, Response to heme deficiency; g, Mitochondrial stress response

As shown in Table 2, 11 of the 12 DEGs associated with the Nrf2-response were strongly increased in RPTEC/TERT1, with the highest relative increases for HMOX1, NMRAL2P and TXNRD1 which were increased more than 20-fold compared to the media controls. The UPR-associated DEGs were increased between 4.5 and 7 fold, whereas the p53-controled GADD45A showed an 11-fold increase of gene expression.

The HepaRG and HUVEC/TERT2 cells showed a lower response of the Nrf2-associated DEGs compared to RPTEC/TERT1-cells. The UPR-response in HepaRG-cells was comparable to that in RPTEC/TERT1 cells, when based on the fold increases of DDIT3, ASNS, TRIB3, TRIM16L and GDF15.

Of the three neuronal cell models, UKN5 was shown to be the least responsive cell to 1,2-DCVC, only showing a 2-fold increase in HMOX1 and NQO1-levels. On the contrary, the BCC42 cells showed relative strong fold increases in DEG levels after exposure to 1,2-DCVC, which in some cases were similar or even higher than those observed in RPTEC/TERT1 cells. BCC42 was the only cell model showing a strong increase of CHAC1, which previously was found to be one of the strongest DEG in 1,2-DCVC-exposed placental cell models (Elkin et al. 2021). The LUHMES cells also showed a significant Nrf2-response and UPR-response. The Nrf2-associated CEBPB gene was increase even more than 100-fold, due to the low expression in media control cells. Also, the UPR-associated TRIB3 gene was increased 70-fold in LUHMES cells, which was the second highest fold increases of all cell models.

The concentration dependence of the fold increases of the 26 DEGs presented in Table 2 can be found in electronic Supplementary file 3, which also shows the concentration-dependent responses of 1,2-DCVG, 2,2-DCVC and 2,2-DCVG. In summary, the following concentration-dependent increases were observed, per cell model:

*RPTEC/TERT1:* These results confirm that RPTEC/TERT1 cells exposed to 1,2-DCVC and 1,2-DCVG showed very similar profiles of DEGs and similar concentration-dependencies. The lowest concentration at which these S-conjugates increased Nrf2-associated DEGs was 3 µM; the 0.3 and 1 µM concentrations did not show any increases higher than 1.5-fold. The UPR-response and p53-signalling responses were significantly increased by 1,2-DCVC after concentration of 30 µM.

2,2-DCVC also showed a clear Nrf2-response in RPTEC/TERT1-cells although at higher concentrations than 1,2-DCVC. HMOX1 expression increased from 1.2-fold at 30 µM up to 16-fold at 500 µM 2,2-DCVC. The corresponding GSH-conjugate 2,2-DCVG displayed comparable responses. No significant activation of the UPR-response and p53-mediated response was observed, even at the highest concentration of 500 µM.

*HepaRG:*as was shown in Table 2, at 62.5 µM 1,2-DCVC HepaRG-cells showed a lower response of Nrf2-associated genes when compared to RPTEC/TERT1-cells. The concentration dependency shows that HMOX1 expression was increased 4-fold at 10 µM, and further increased to 28-fold at 125 µM. The UPR responses and p53-mediated responses were only significantly activated by 1,2-DCVC at 62.5 µM and higher.

2,2-DCVC showed to activate the Nrf2-pathway in HepaRG-cells at concentrations of 125 µM or higher. The UPR and p53-associated genes were only increased by 2,2-DCVC 2-fold at 500 µM.

*HUVEC/TERT2:* the first Nrf2-responses in HUVEC/TERT2 were observed at 10 µM 1,2-DCVC where increases of HMOX1 (1.6x) and NQO1 (1.5x) were found. A further increase was observed up to 7.3-fold (HMOX1) and 3.6-fold (NQO1) at 125 µM. At this concentration, NMRAL2P expression was increased 11-fold, relative to media-control. 1,2-DCVG showed similar responses as 1,2-DCVC but starting at a higher concentration of 30 µM. 2,2-DCVC only caused relatively mild Nrf2-response at 250 and 500 µM.

The UPR-associated DEGs and p53-associated genes were slightly (< twofold) increased at concentrations of 62.5 and 125 µM of 1,2-DCVC. No increases of these stress responses were observed after exposure to the highest concentrations of 1,2-DCVG, 2,2-DCVC and 1,2-DCVG.

*LUHMES:* LUHMES showed significant Nrf2-responses starting from 10 µM 1,2-DCVC, with a 5-fold increase in NQO1-expression. The UPR-response was only increased at 62.5 µM 1,2-DCVC.

The corresponding GSH-conjugate 1,2-DCVG and the 2,2-regioisomers did not show a more than 1.5-fold increase in DEGs associated with Nrf2, UPR or p53-signalling pathway.

*BCC42:* as shown in Table 2, BCC42 cells showed several large stress responses after exposure to 1,2-DCVC which were concentration dependent, Online Resources 5—Supplemental Table S4. At 30 µM 1,2-DCVC the first increases in Nrf2 and UPR-responses were observed. P53-dependent response was observed at 62.5 µM 1,2-DCVC and higher. 1,2-DCVG only showed Nrf2-response at 250 and 500 µM; no effect was observed on the UPR and p53-dependent stress response.

The regioisomer 2,2-DCVC only caused a relative weak Nrf2-response at 250 and 500 µM. No effects were observed when exposing BCC42 cells to 2,2-DCVG at concentrations up to 500 µM.

*UKN5-cells:* as already shown in Table 2, the UKN5-cell showed the lowest response after exposure to 62,5 µM 1,2-DCVC. Increasing the concentration to 125 µM only showed a small increase in expression of HMOX1 (2.9x) and NQO1 (3.8x). At this concentration also relatively small increases in UPR-associated DEGs were found: DDIT3 (2,3x), TRIB3 (4.1x) and ASNS (2.2x). The expression of CHAC1 was increased 2.5-fold at 125 µM 1,2-DCVC.

Exposure of UKN5 cells to regioisomer 2,2-DCVC did not result in any changes higher than 1.5-fold. Since the exposure to the cysteine conjugates lead to the low responses in UKN5, no experiments were performed with the corresponding GSH-conjugates.

### Basal expression of genes coding enzymes involved in DCVG and DCVC metabolism

**Fig. 7:**
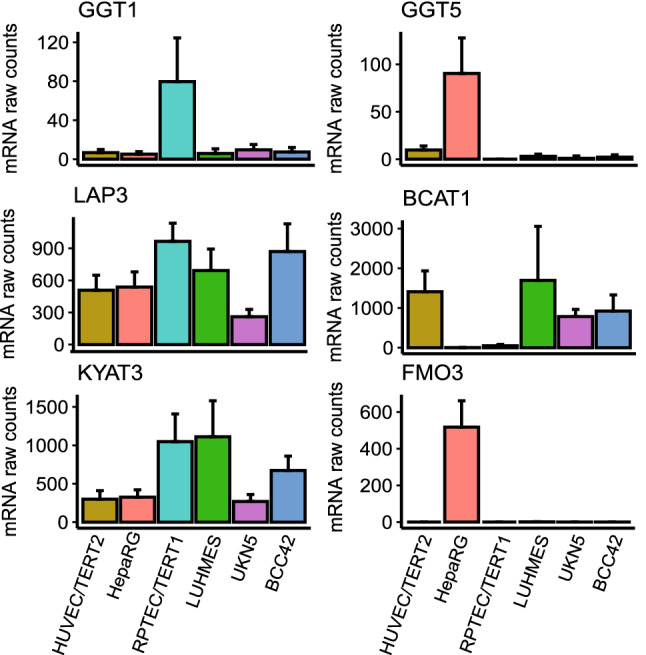
mRNA baseline expression of untreated samples for genes potentially involved in the biotransformation of TCE after glutathione conjugation. GGT activity: γ-glutamyltransferase (GGT1) and γ-glutamyl leukotrienase (GGT5), Dipeptidase activity: cysteinylglycine-S-Conjugate Dipeptidase (LAP3), β-lyase activity: kynurenine aminotransferase 3 (KYAT3), branched chain amino acid transaminase 1 (BCAT1) and FMO activity: flavin containing dimethylaniline monoxygenase 3 (FMO3). Genes are colour coded by cell model as shown in Fig. 2

The basal gene expression of enzymes potentially involved in the biotransformation of DCVG and DCVC was assessed in media control samples (Fig. 7). The hydrolysis of the γ-glutamyl group of DCVG can be catalyzed by GGT1 and GGT5. RPTEC/TERT1 showed the highest expression of GGT1, whereas GGT5 was found at highest levels in HepaRG. Expression of cysteinylglycine-S-conjugate dipeptidase (LAP3) was found in all tested cells. KYAT3, one of the human β-lyases active in bioactivation of 1,2-DCVC (Pinto et al. 2014), showed the highest expression levels in RPTEC/TERT1 and LUHMES. BCAT1, another identified human β-lyase enzyme (Cooper and Pinto 2006) seems to be not expressed in HepaRG and RPTEC/TERT1 but showed significant expression in the other four cell models. Expression of flavin-containing mono-oxygenase 3 (FMO3), which is proposed to be involved in bioactivation of DCVC by sulphoxidation (Cristofori et al. 2015) was found exclusively in HepaRG-cells.

## Discussion

In the present study, six human cell models were evaluated to explore tissue selective molecular effects of GSH-derived S-conjugates of TCE. Based on a large number of in vitro studies and in vivo studies using proximal tubular cells from various mammalian species, it is generally accepted that 1,2-DCVC plays an important role in the nephrotoxic effects of TCE in humans (Cichocki et al. 2016). More recently, it has been speculated that 1,2-DCVC may also play a role in other extrahepatic effects, such as birth defects and neuronal effects (Elkin et al. 2021; De Miranda and Greenamyre 2020). The panel of well characterized cell lines and novel hiPSC derived models selected are representative of four tissues/organs: RPTEC/TERT1 as proximal tubular model, HepaRG as liver model, HUVEC/TERT2 as vascular endothelial model, and LUHMES, UKN5 and BCC42 as neuronal models. These in vitro systems were found to have specific transcriptomic signatures and express tissue-specific cell markers, suggesting their suitability as tissue/organ representatives (Fig. 2).

### Evaluation of in vitro models representing target tissues of TCE S-conjugates

In our study, 1,2-DCVC affected the cell viability of the six human cell models tested to a different extent (Fig. 3). The TempO-Seq assay demonstrated that all the cell models from the panel were sensitive to 1,2-DCVC exposure in a concentration-dependent manner (except for UKN5 where only two concentrations were tested). The transcriptomic changes found did not reflect entirely the sensitivity levels from the viability assessments (Fig. 4).

#### RPTEC/TERT1

The RPTEC/TERT1 cells has been proposed as a model system for the human proximal tubular cells and has been shown to be sensitive to different known nephrotoxicants (Aschauer et al. 2015; Limonciel et al. 2018). The present study, for the first time demonstrates that this cell model shows high sensitivity to both 1,2-DCVC and 1.2-DCVG, which have been shown to be cytotoxic in proximal tubular cells isolated from rat and human kidneys (Lash et al. 2005; Lock et al. 2006). The fact that 1,2-DCVG showed almost the same effects on viability and cellular responses as 1,2-DCVC is in line with observation that both conjugates showed similar effects in viability (MTT-activity) in exposed human proximal tubular cells (Lock et al. 2006). The similar effects in RPTEC/TERT1 indicates that this renal cell model expresses a high level of GGT, a well-known characteristic of proximal tubular cells. These results indicate the RPTEC/TERT1-cells are excellent in vitro models to study cellular responses to the GSH-derived metabolites of not only TCE but also to less well studied halogenated compounds. In addition, GSH-conjugates of a variety of polyphenols are nephrotoxicants in a variety of animal models, which was shown to be dependent on the relatively high activity of GGT (Bolton et al. 2000). Although isolated proximal tubular cells from human kidney showed sensitivity towards 1,2-DCVC, it was observed that large differences in viability and cellular responses were observed between cell isolated from different donors (Lock et al. 2006). This interindividual variability may result from genetic polymorphisms or epigenetic factors affecting the activities of enzymes and transporters involved in processing the GSH-conjugates. Next to the limited availability of human kidney material, this variability strongly limits their use for mechanistic studies and regulatory applications. The use of RPTEC/TERT1-cells which is a well-defined model will produce more reproducible results and will allow standardization of experimental conditions.

#### Neuronal cell models (LUHMES, UKN5 and BCC42)

Based on animal and epidemiologic studies, exposure to TCE has been associated with several neurotoxic effects in both the central and peripheral nervous system. While the exact mechanisms underlying the neurotoxic effects of TCE remains to be established, it has been hypothesized that bioactivation by the GSH-conjugation pathway might play a role in the pathogenesis of several neurotoxic effects of TCE. Formation of 1,2-DCVG and/or 1,2-DCVG has also been proposed to underly the neurotoxic effects of dichloroacetylene, which is conjugated to GSH at much higher rate than TCE (Dekant et al. 1989; Patel et al. 1993, 1994). Epidemiological studies suggest that exposed workers may be at an increased risk of Parkinson’s disease. Animal studies have supported the link between TCE and Parkinson’s disease by the observation that TCE exposure cause significant damage to loss of dopamine neurons in the nigrostriatal dopamine system (Gash et al. 2008).

The LUHMES cells used in the present study are a model for human dopaminergic neurons and have been used to study mechanisms of neural degeneration underlying Parkinson’s disease (Tong et al. 2022). The present study demonstrates that the viability of LUHMES is significantly decreased by 1,2-DCVC, almost to the same extent as RPTEC/TERT1 (Fig. 3a). LUHMES show expression of two β-lyases (KYAT3 and BCAT1) that are active in bioactivation of 1,2-DCVC (Cooper and Pinto 2006). 1,2-DCVG did not cause effects, which might be explained by the low GGT-expression (Fig. 3a). Recently it was demonstrated that LUHMES dopaminergic neurons are highly susceptible to ferroptosis, a form of necrotic cell death caused by lipid peroxidation (Tong et al. 2022). Interestingly, ferroptosis was one of the 1,2-DCVC-induced stress responses identified by the KEGG pathway source, Table 1. Therefore, this is the first mechanistic study supporting for recent hypothesis that the GSH-conjugation might play a role in the pathogenesis of TCE-induced Parkinson’s disease.

BCC42 neuronal cells showed only a small 1,2-DCVC-induced decrease in viability when compared to LUHMES and UKN5-cells (Fig. 3a). Interestingly, a relatively strong Nrf2-response was observed, indicative for the internal exposure to β-lyase mediated reactive metabolites.

#### HUVEC/TERT2

HUVEC/TERT2 is a cell line derived from human umbilical vein endothelial cells. This cell-line showed only a small decrease in cell viability after exposure to high concentration of 1,2-DCVC (Fig. 3a) and only small increases in gene expression belonging to the Nrf2 and UPR stress responses (Fig. 6). 1,2-DCVG was without effects due to the low GGT-expression. Although the plasma-concentration of DCVC and DCVG remain to be established, the fact that effects only occurred at high concentrations of 1,2-DCVC suggest that cytotoxicity to umbilical vein endothelial cells is unlikely to contribute to the birth defects associated with TCE-exposure. Toxic effects of 1,2-DCVC towards placental cells, as shown recently, (Elkin et al. 2018; Elkin et al. 2019; Elkin et al. 2021) using placental cell models, are therefore more likely to be involved in TCE-induced birth defects.

#### HepaRG

HepaRG has emerged as a promising in vitro model representing human hepatocytes to study mechanisms of hepatotoxicants requiring oxidative bioactivation, since after DMSO-triggered differentiation, it shows expression levels of P450s comparable to isolated human hepatocytes (Biopredic 2017). Although in vivo animal studies demonstrated that DCVC does not target the liver, isolated rat hepatocytes appeared to be sensitive to 1,2-DCVC-toxicity, more than the oxidative metabolites of TCE (trichloroacetate, dichloroacetate, chloral hydrate, trichloroethanol) (Lash et al. 1995; Lash and Parker 2001). The present study shows that exposure of HepaRG-cells to 1,2-DCVC resulted in decreased cell viability and activation of several stress responses, Figs. 3a, 4a and 5a. These results can be explained by the hepatic expression of human β-lyase KYAT3 (Fig. 7).

### Comparison of transcriptomic responses caused by 1,2-DCVC and 1,2-DCVG in cellular models

So far only two studies have focussed on the early transcriptomic responses caused by 1,2-DCVC in human target cells (Lock et al. 2006; Elkin et al. 2021). Lock et al. (2006) used human renal proximal tubule cells from four individuals which were exposed to two low concentrations (0.1 and 1 µM) of 1,2-DCVC for 10 days. The results showed that these low concentrations resulted in only relatively small changes (mostly < 2-fold) in expression of gene products associated with apoptosis, oxidative stress and cell proliferation. However, large interindividual differences were observed: some changes were only observed in 2 out of the 4 samples. More recently, the transcriptomic responses were studied in HTr-8/SVneo human trophoblast cell line exposed for 6 and 12 h to 1,2-DCVC (10 and 20 µM) (Elkin et al. 2021). This placental model was selected to study the possible role of DCVC in birth defects caused by TCE and showed that DCVC caused an integrated stress response (ISR) activated by the ATF4 transcription factor. In the present study, the transcriptomic responses caused by 1,2-DCVC in six cell models representing potential target tissue while using a wider range of concentrations (0,03–500 µM) are comparable to the studies mentioned above.

As summarized in Table 1 and Fig. 5, a wide variety of transcriptomic responses were observed after 1,2-DCVC exposure representing multiple stress response pathways. Over-representation analysis showed that the most prominent stress response pathways at both low and high concentrations was the *Nrf2* pathway, which represents a cellular response to oxidative and/or electrophilic stress*.* At higher concentrations of 1,2-DCVC, UPR, Inflammation and DNA damage response were found in addition. In our study, genes belonging to *Nrf2* (e.g., HMOX1, GCLM, FTL) and UPR (e.g., TRIB3, DDIT3, ASNS) were found to be commonly differentially expressed among all six cell models exposed to the highest sub-cytotoxic concentrations of 1,2-DCVC (Fig. 5a). The different degree of upregulation observed between the cell models, Fig. 6, may be related to the difference in activities of uptake transporters and β-lyase bioactivation, leading to different intracellular exposure to the reactive thioketene. Kinetic analysis of rates of cellular uptake and bioactivation is required to support this hypothesis.

After 1,2-DCVG exposure, only RPTEC/TERT1, HepaRG, HUVEC/TERT2 and BCC42 were responsive whereas the other cells were non-responsive to 1,2-DCVG, which may be attributed by the absence or very low activity of GGT. The cells responsive to 1,2-DCVG were mainly from the *Nrf2* pathway and no UPR genes were found, Fig. 5b.

Comparison of the transcriptomic profiles found in the present study with those of Lock and Elkin et al., is hampered by the fact that different concentrations and exposure times of 1,2-DCVC were used, next to different methodologies of transcriptomic analysis. Several similarities in genes expression were observed in our study and the study of Elkin et al. using placental cell model (Elkin et al. 2021). For example, both studies found upregulation of gene expression of ASNS, TRIB3 and DDIT3 indicative for the UPR stress response. In contrast, less than 1.3-fold increases of expression of HMOX1, GCLM, FTL, GADD45A, MDM2 and SNAI2 were observed in the placental model. A side by side  comparison of the changes in expression of 26 genes observed in the present and Elkin's study (2021) can be found in electronic Supplementary file 4.

### Comparison of effects of regioisomers of DCVC and DCVG

Recently, we demonstrated that 2,2-DCVG was the major GSH-conjugate formed in incubations of TCE with human liver fractions (Capinha et al. 2021). Therefore, a contribution of the 2,2-regioisomers to adverse effects in human tissues should be considered. So far, only four studies have investigated the difference in toxicity of 1,2-DCVC and 2,2-DCVC in rat and mouse models (Commandeur et al. 1991; Birner et al. 1997; Ilinskaja and Vamvakas 1996). The present study demonstrates that the higher toxicity of the 1,2-regioisomers also applies to human cells. The 2,2-regioisomers of DCVC and DCVG showed much smaller effects on cell viability and stress responses in all cell models when compared to the 1,2-regioisomers (Figs. 3, 4 and 6). As a possible explanation for these different effects, formation of different types of reactive intermediate has been proposed. As shown in Fig. 1, 1,2-DCVC is converted to a reactive thioketene which has cross-linking properties, whereas 2,2-DCVC is converted to a less reactive thioaldehyde. Despite the low to absent effects of 2,2-DCVG and 2,2-DCVC across the six models (up to 500 µM), a small number of concentration-dependent transcriptomic changes were found in RPTEC/TERT1, HUVEC/TERT2 and HepaRG after 2,2-DCVC exposure. These results showed that despite the low reactivity of the thioaldehyde-product of 2,2-DCVC, some transcriptomic changes were triggered.

## Conclusion

The present study, utilising TCE GSH conjugation products, demonstrates that the utilization of human cells from different tissues (target and non-target) in combination with transcriptomics can provide tissue-specific effects to confirm and further elaborate molecular mechanisms of toxicity. The cell models with the highest expression of mRNA encoding GGT enzymes, showed strong transcriptomic responses to both 1,2-DCVG and 1,2-DCVC. RPTEC/TERT1 was the most affected model to both 1,2-DCVG and 1,2-DCVC. Exposure to 2,2-DCVG and 2,2-DCVC also resulted in oxidative stress responses in this cell model, but at higher exposure concentrations. The stress responses induced in two neuronal models, LUHMES and BCC42, also suggest that 1,2-DCVC may be involved in the hypothesized neurotoxic effects of TCE. This study expands our knowledge on the mode of action of TCE toxicity via GSH conjugation pathway and demonstrates the usefulness of combining a panel of human cell systems with transcriptomics to delineate the molecular events responsible for tissue specificity of analogue compounds.

## Supplementary Information

Below is the link to the electronic supplementary material.

Supplementary file1 (Flow chart) (DOCX 34 KB)

Supplementary file2 (Pathway analysis) (XLSX 41 KB)

Supplementary file3 (DRCs 26 DEGs) (XLSX 75 KB)

Supplementary file4 (Comparison data Elkin) (DOCX 40 KB)

Supplementary file5 (TempoSeq HUVEC/TERT2) (XLSX 8194 KB)

Supplementary file6 (TempoSeq HepaRG) (XLSX 8683 KB)

Supplementary file7 (TempoSeq RPTEC/TERT1) (XLSX 8268 KB)

Supplementary file8 (TempoSeq LUHMES) (XLSX 2849 KB)

Supplementary file9 (TempoSeq UKN5) (XLSX 1049 KB)

Supplementary file10 (TempSeq BCC42) (XLSX 8705 KB)

Supplementary file11 (DEGs HUVEC/TERT2) (XLSX 38 KB)

Supplementary file12 (DEGs HepaRG) (XLSX 375 KB)

Supplementary file13 (DEGs RPTEC/TERT1) (XLSX 153 KB)

Supplementary file14 (DEGs LUHMES) (XLSX 30 KB)

Supplementary file15 (DEGs UKN5) (XLSX 18 KB)

Supplementary file16 (DEGs BCC42) (XLSX 69 KB)

## Data Availability

The data supporting the findings in this study are available in its Supplementary Information. Raw data are available from the corresponding author, upon reasonable request.
