# Supplementary material for: Transcriptomic-based evaluation of trichloroethylene glutathione and cysteine conjugates demonstrate phenotype-dependent stress responses in a panel of human in vitro models
Source: Arch Toxicol. 2022 Dec 28;97(2):523–45. doi: 10.1007/s00204-022-03436-6 (PMC9859926; doi:10.1007/s00204-022-03436-6)
Supplement: Supplementary file 1 — Supplementary file1 (Flow chart) (DOCX 34 KB) [file 204_2022_3436_MOESM1_ESM.docx]

Transcriptomic-based evaluation of trichloroethylene glutathione and cysteine conjugates demonstrates phenotype-dependent stress responses in a panel of human in vitro models.

Liliana Capinha^1^*, Yaran Zhang^1,2^, Anna-Katharina Holzer^3^, Anna-Katharina Ückert^3^, Melinda Zana^4^, Giada Carta^1^, Cormac Murphy^1^, Jenna Baldovini^1^, Zahra Mazidi^5^, Johannes Grillari^5^, Andras Dinnyes^4,6^, Bob van der Water^7^, Marcel Leist^3^, Jan N. M. Commandeur^1^ and Paul Jennings^1^

*Affiliations*

*^1^ Division of Molecular and Computational Toxicology, Amsterdam Institute for Molecules, Medicines and Systems, Vrije Universiteit Amsterdam, De Boelelaan 1108, 1081 HZ Amsterdam, The Netherlands*

*^2^ Genomics of Neurodegenerative Diseases and Aging, Human Genetics, Vrije Universiteit Amsterdam, Amsterdam UMC location VUmc, Amsterdam, The Netherlands*

*^3^In Vitro Toxicology and Biomedicine, Dept Inaugurated by the Doerenkamp-Zbinden Foundation, University of Konstanz, 78457 Konstanz, Germany
^4^ BioTalentum Ltd, Godollo, 2100, Hungary*

*^5^ Evercyte GmbH, Vienna, Austria; Institute of Molecular Biotechnology, Department of Biotechnology, BOKU - University of Natural Resource and Life science (BOKU), Vienna, Austria.*

*^6^Hungarian University of Agriculture and Life Sciences, Institute of Physiology and Animal Nutrition, Department of Physiology and Animal Health, Gödöllő, 2100 Hungary*

*^7^Division of Drug Discovery and Safety, Leiden Academic Centre for Drug Research (LACDR), Leiden University, Leiden, The Netherlands.*

***Corresponding author
Liliana Capinha, [l.santoscapinha@vu.nl](mailto:l.santoscapinha@vu.nl)

Sample passed quality check?

Count matrix

Yes

No

Keep sample

Remove sample

Modified count matrix

Modified count matrix of media samples of all cell models

Regularized log transformation

Principal component analysis

Differential expression analysis of each condition (DEGs) per cell type

Over-representation pathway analysis

mRNA counts dose response of specific genes

mRNA raw count gene expression cell makers/enzymes

**Figure S1.** Flowchart describing transcriptomic (TempO-Seq) data processing from count matrix (mRNA read counts).
