# Supplementary material for: Transcriptomic-based evaluation of trichloroethylene glutathione and cysteine conjugates demonstrate phenotype-dependent stress responses in a panel of human in vitro models
Source: Arch Toxicol. 2022 Dec 28;97(2):523–45. doi: 10.1007/s00204-022-03436-6 (PMC9859926; doi:10.1007/s00204-022-03436-6)
Supplement: Supplementary file 4 — Supplementary file4 (Comparison data Elkin) (DOCX 40 KB) [file 204_2022_3436_MOESM4_ESM.docx]

Transcriptomic-based evaluation of trichloroethylene glutathione and cysteine conjugates demonstrates phenotype-dependent stress responses in a panel of human in vitro models.

Liliana Capinha^1^*, Yaran Zhang^1,2^, Anna-Katharina Holzer^3^, Anna-Katharina Ückert^3^, Melinda Zana^4^, Giada Carta^1^, Cormac Murphy^1^, Jenna Baldovini^1^, Zahra Mazidi^5^, Johannes Grillari^5^, Andras Dinnyes^4,6^, Bob van der Water^7^, Marcel Leist^3^, Jan N. M. Commandeur^1^ and Paul Jennings^1^

*Affiliations*

*^1^ Division of Molecular and Computational Toxicology, Amsterdam Institute for Molecules, Medicines and Systems, Vrije Universiteit Amsterdam, De Boelelaan 1108, 1081 HZ Amsterdam, The Netherlands*

*^2^ Genomics of Neurodegenerative Diseases and Aging, Human Genetics, Vrije Universiteit Amsterdam, Amsterdam UMC location VUmc, Amsterdam, The Netherlands*

*^3^In Vitro Toxicology and Biomedicine, Dept Inaugurated by the Doerenkamp-Zbinden Foundation, University of Konstanz, 78457 Konstanz, Germany
^4^ BioTalentum Ltd, Godollo, 2100, Hungary*

*^5^ Evercyte GmbH, Vienna, Austria; Institute of Molecular Biotechnology, Department of Biotechnology, BOKU - University of Natural Resource and Life science (BOKU), Vienna, Austria.*

*^6^Hungarian University of Agriculture and Life Sciences, Institute of Physiology and Animal Nutrition, Department of Physiology and Animal Health, Gödöllő, 2100 Hungary*

*^7^Division of Drug Discovery and Safety, Leiden Academic Centre for Drug Research (LACDR), Leiden University, Leiden, The Netherlands.*

***Corresponding author
Liliana Capinha, [l.santoscapinha@vu.nl](mailto:l.santoscapinha@vu.nl)

**Supplemental Table S5a.**  Comparison of fold increases of a selection of differentially expressed genes in the cell models from our study exposed for 24h to 10 µM of 1,2-DCVC and placental models (HTR-8/SVneo and Villous explants) exposed for 12 h to 20 µM of 1,2-DCVC.

| Differentially expressed genes | Associated stress pathway(s) | **RPTEC/ TERT1** | **HepaRG** | **HUVEC/ TERT2** | **LUHMES** | **BBC42** | **HTR-8/ SVneo *)** | **Villous explants *)** |
| --- | --- | --- | --- | --- | --- | --- | --- | --- |
| [1,2-DCVC] |  | 9.3 µM | 9.3 µM | 9.3 µM | 9.3 µM | 9.3 µM | 20 µM | 20 µM |
| *HMOX1* | *a, d* | **5.6** | 3.9 | 1.6 | < 1.5 | < 1.5 | 1.5 | 1.4 |
| *GCLM* | *a, d* | **2.5** | 1.7 | < 1.5 | < 1.5 | < 1.5 | < 1.3 | 1.7 |
| *FTL* | *a, d* | **2.2** | < 1.5 | < 1.5 | < 1.5 | < 1.5 | 1.3 | 1.3 |
| *UGT1A6* | *a, e* | **7.1** | < 1.5 | < 1.5 | < 1.5 | < 1.5 | n.t. | n.t. |
| *UGT1A8* | *a* | **7.7** | < 1.5 | < 1.5 | < 1.5 | < 1.5 | n.t. | n.t. |
| *UGT1A10* | *a* | **6** | < 1.5 | < 1.5 | < 1.5 | < 1.5 | n.t. | n.t. |
| *SRXN1* | *a* | 2 | **2.5** | < 1.5 | < 1.5 | < 1.5 | < 1.3 | 1.5 |
| *CEBPB* | *a* | < 1.5 | < 1.5 | < 1.5 | < 1.5 | < 1.5 | 1.9 | < 1.3 |
| *SLC7A11* | *a* | < 1.5 | **2.9** | < 1.5 | < 1.5 | < 1.5 | 2.4 | 1.8 |
| *NMRAL2P* | *a* | 4.5 | < 1.5 | < 1.5 | < 1.5 | < 1.5 | n.t. | n.t. |
| *OSGIN1* | *a* | < 1.5 | < 1.5 | < 1.5 | < 1.5 | < 1.5 | < 1.3 | 2.4 |
| *NQO1* | *a* | 4.6 | < 1.5 | 1.5 | **5.1** | < 1.5 | 1.4 | < 1.3 |
| *TXNRD1* | *a, e* | **5.3** | < 1.5 | < 1.5 | < 1.5 | < 1.5 | 1.5 | 1.6 |
| *DDIT3* | *b* | < 1.5 | < 1.5 | < 1.5 | < 1.5 | < 1.5 | 1.7 | 1.9 |
| *ASNS* | *b* | < 1.5 | < 1.5 | < 1.5 | < 1.5 | < 1.5 | < 1.3 | 1.3 |
| *TRIB3* | *b* | < 1.5 | < 1.5 | < 1.5 | < 1.5 | < 1.5 | 2.1 | < 1.3 |
| *TRIM16L* | *b* | **3.2** | < 1.5 | < 1.5 | < 1.5 | 2.6 | 2.3 | < 1.3 |
| *GDF15* | *b, g* | < 1.5 | **1.9** | < 1.5 | < 1.5 | < 1.5 | < 1.3 | < 1.3 |
| *GADD45A* | *c* | < 1.5 | < 1.5 | < 1.5 | < 1.5 | < 1.5 | 1.5 | 1.6 |
| *SNAI2* | *c* | < 1.5 | < 1.5 | < 1.5 | < 1.5 | < 1.5 | < 1.3 | < 1.3 |
| *MDM2* | *c* | 1.7 | < 1.5 | < 1.5 | < 1.5 | < 1.5 | < 1.3 | < 1.3 |
| *TNFRSF10B* | *c* | < 1.5 | < 1.5 | < 1.5 | **2.8** | < 1.5 | < 1.3 | 1.6 |
| *SLC7A5* | *e* | < 1.5 | < 1.5 | < 1.5 | < 1.5 | < 1.5 | < 1.3 | < 1.3 |
| *ME1* | *e* | < 1.5 | < 1.5 | < 1.5 | **2.9** | < 1.5 | 1.4 | < 1.3 |
| *CHAC1* | *f* | < 1.5 | < 1.5 | < 1.5 | < 1.5 | < 1.5 | **5.8** | **1.7** |
| *ATF5* | *f* | < 1.5 | < 1.5 | < 1.5 | < 1.5 | < 1.5 | < 1.3 | < 1.3 |

Numbers represent fold-increases of DEGs relative to the medium controls; numbers in bold represent the largest fold increase across the six cell models;

a. Nrf2-response; b. UPR-response; c. p53-signalling; d. Ferroptosis; e. Nuclear receptor Meta-pathway; f. Response to heme deficiency; g. Mitochondrial stress response.
*Elkin et al. 2021

**Table S5b.**  Comparison of fold increases of a selection of differentially expressed genes in the cell models from our study exposed for 24h to 30 µM of 1.2-DCVC and placental models (HTR-8/SVneo and Villous explants) exposed for 12 h to 20 µM of 1.2-DCVC..

| Differentially expressed genes | Associated stress pathway(s) | **RPTEC/ TERT1** | **HepaRG** | **HUVEC/ TERT2** | **LUHMES** | **BBC42** | **HTR-8/ SVneo *)** | **Villous explants *)** |
| --- | --- | --- | --- | --- | --- | --- | --- | --- |
| [1.2-DCVC] |  | 30 µM | 30 µM | 30 µM | 30 µM | 30 µM | 20 µM | 20 µM |
| *HMOX1* | *a, d* | **18** | 9.5 | 2.7 | 2.3 | 5.9 | 1.5 | 1.4 |
| *GCLM* | *a, d* | **4.7** | 2 | 1.5 | **2** | 2.9 | < 1.3 | 1.7 |
| *FTL* | *a, d* | **4.9** | 1.8 | < 1.5 | 2.1 | 1.9 | 1.3 | 1.3 |
| *UGT1A6* | *a, e* | **11** | < 1.5 | < 1.5 | < 1.5 | < 1.5 | n.t. | n.t. |
| *UGT1A8* | *a* | **9.1** | < 1.5 | < 1.5 | < 1.5 | < 1.5 | n.t. | n.t. |
| *UGT1A10* | *a* | **8.7** | < 1.5 | < 1.5 | < 1.5 | < 1.5 | n.t. | n.t. |
| *SRXN1* | *a* | 3.5 | 3.4 | 1.6 | < 1.5 | **6.7** | < 1.3 | 1.5 |
| *CEBPB* | *a* | < 1.5 | < 1.5 | < 1.5 | < 1.5 | **3.5** | 1.9 | < 1.3 |
| *SLC7A11* | *a* | < 1.5 | **4.8** | 2.3 | < 1.5 | 3 | 2.4 | 1.8 |
| *NMRAL2P* | *a* | **20** | < 1.5 | 5.4 | < 1.5 | 17 | n.t. | n.t. |
| *OSGIN1* | *a* | 9 | 3.4 | < 1.5 | < 1.5 | **17** | < 1.3 | 2.4 |
| *NQO1* | *a* | **8.2** | < 1.5 | 2.2 | 6.8 | 3 | 1.4 | < 1.5 |
| *TXNRD1* | *a, e* | **5.2** | < 1.5 | 1.9 | < 1.5 | < 1.5 | 1.5 | 1.6 |
| *DDIT3* | *b* | < 1.5 | 1.9 | < 1.5 | < 1.5 | **3.3** | 1.7 | 1.9 |
| *ASNS* | *b* | 1.7 | < 1.5 | < 1.5 | < 1.5 | **2.5** | < 1.3 | 1.3 |
| *TRIB3* | *b* | < 1.5 | < 1.5 | < 1.5 | < 1.5 | **3.1** | 2.1 | < 1.3 |
| *TRIM16L* | *b* | **7.1** | 1.6 | 2.6 | 2 | 5 | 2.3 | < 1.3 |
| *GDF15* | *b, g* | 2.5 | 3 | < 1.5 | 7 | **3.2** | < 1.3 | < 1.3 |
| *GADD45A* | *c* | **2.2** | < 1.5 | < 1.5 | < 1.5 | < 1.5 | 1.5 | 1.6 |
| *SNAI2* | *c* | **2.3** | < 1.5 | < 1.5 | < 1.5 | < 1.5 | < 1.3 | < 1.3 |
| *MDM2* | *c* | **1.6** | < 1.5 | **1.6** | < 1.5 | < 1.5 | < 1.3 | < 1.3 |
| *TNFRSF10B* | *c* | < 1.5 | < 1.5 | < 1.5 | **2.9** | < 1.5 | < 1.3 | 1.6 |
| *SLC7A5* | *e* | 1.9 | **3.2** | 3.1 | < 1.5 | < 1.5 | < 1.3 | < 1.3 |
| *ME1* | *e* | < 1.5 | < 1.5 | < 1.5 | **3.8** | < 1.5 | 1.4 | < 1.3 |
| *CHAC1* | *f* | < 1.5 | < 1.5 | < 1.5 | < 1.5 | **6.7** | 5.8 | 1.7 |
| *ATF5* | *f* | < 1.5 | < 1.5 | < 1.5 | < 1.5 | **2.8** | < 1.3 | < 1.3 |

Numbers represent fold-increases of DEGs relative to the medium controls; numbers in bold represent the largest fold increase across the six cell models;

a. Nrf2-response; b. UPR-response; c. p53-signalling; d. Ferroptosis; e. Nuclear receptor Meta-pathway; f. Response to heme deficiency; g. Mitochondrial stress response.

*Elkin et al. 2021

**Table S5c.**  Comparison of fold increases of a selection of differentially expressed genes in the cell models from our study exposed for 24h to 62.5 µM of 1.2-DCVC and placental models (HTR-8/SVneo and Villous explants) exposed for 12 h to 20 µM of 1.2-DCVC.

| Differentially expressed genes | Associated stress pathway(s) | **RPTEC/ TERT1** | **HepaRG** | **HUVEC/ TERT2** | **LUHMES** | **BBC42** | **UKN5** | **HTR-8/ SVneo *)** | **Villous explants *)** |
| --- | --- | --- | --- | --- | --- | --- | --- | --- | --- |
| [1.2-DCVC] |  | 62.5 µM | 62.5 µM | 62.5 µM | 62.5 µM | 62.5 µM | 62.5 µM | 20 µM | 20 µM |
| *HMOX1* | *a, d* | 23 | 12 | 3.9 | 22 | **35** | 2.0 | 1.5 | 1.4 |
| *GCLM* | *a, d* | 6.0 | 2.4 | 1.9 | **8.1** | 6.1 | <1.5 | < 1.3 | 1.7 |
| *FTL* | *a, d* | **4.6** | 2.5 | 1.7 | 4.1 | 3.9 | < 1.5 | 1.3 | 1.3 |
| *UGT1A6* | *a, e* | **8.7** | < 1.5 | < 1.5 | < 1.5 | < 1.5 | < 1.5 | n.t. | n.t. |
| *UGT1A8* | *a* | **7.8** | < 1.5 | < 1.5 | < 1.5 | < 1.5 | < 1.5 | n.t. | n.t. |
| *UGT1A10* | *a* | **7.9** | < 1.5 | < 1.5 | < 1.5 | < 1.5 | < 1.5 | n.t. | n.t. |
| *SRXN1* | *a* | 7.0 | 4.6 | 1.7 | < 1.5 | **21** | < 1.5 | < 1.3 | 1.5 |
| *CEBPB* | *a* | 3.0 | < 1.5 | 1.6 | **112** | 4.2 | < 1.5 | 1.9 | < 1.3 |
| *SLC7A11* | *a* | < 1.5 | **7.7** | 2.7 | < 1.5 | 5.6 | < 1.5 | 2.4 | 1.8 |
| *NMRAL2P* | *a* | 25 | < 1.5 | 9.2 | < 1.5 | **33** | < 1.5 | n.t. | n.t. |
| *OSGIN1* | *a* | 14 | 3.9 | 2.3 | < 1.5 | **30** | < 1.5 | < 1.3 | 2.4 |
| *NQO1* | *a* | 8.4 | < 1.5 | 2.6 | **15** | 4.4 | 2.3 | 1.4 | < 1.5 |
| *TXNRD1* | *a, e* | **21** | < 1.5 | 1.9 | 3.0 | 4.3 | < 1.5 | 1.5 | 1.6 |
| *DDIT3* | *b* | 7.7 | 4.4 | < 1.5 | **17** | 5.8 | < 1.5 | 1.7 | 1.9 |
| *ASNS* | *b* | 5.3 | 3.1 | < 1.5 | **7.8** | 4.0 | < 1.5 | < 1.3 | 1.3 |
| *TRIB3* | *b* | 4.5 | 5.3 | < 1.5 | **70** | 10 | < 1.5 | 2.1 | < 1.3 |
| *TRIM16L* | *b* | 6.6 | 1.5 | 3.8 | 6.3 | **8.1** | < 1.5 | 2.3 | < 1.3 |
| *GDF15* | *b, g* | 7.0 | 5.7 | < 1.5 | 11 | **16** | < 1.5 | < 1.3 | < 1.3 |
| *GADD45A* | *c* | **11** | 1.8 | < 1.5 | < 1.5 | 5.7 | < 1.5 | 1.5 | 1.6 |
| *SNAI2* | *c* | **4.3** | 2.1 | < 1.5 | < 1.5 | < 1.5 | < 1.5 | < 1.3 | < 1.3 |
| *MDM2* | *c* | < 1.5 | < 1.5 | 1.6 | < 1.5 | **5.1** | < 1.5 | < 1.3 | < 1.3 |
| *TNFRSF10B* | *c* | 1.9 | < 1.5 | < 1.5 | **4.1** | 3.0 | < 1.5 | < 1.3 | 1.6 |
| *SLC7A5* | *e* | 4.7 | 5.1 | 5.5 | **40** | < 1.5 | < 1.5 | < 1.3 | < 1.3 |
| *ME1* | *e* | 1.8 | < 1.5 | < 1.5 | **13** | < 1.5 | < 1.5 | 1.4 | < 1.3 |
| *CHAC1* | *f* | < 1.5 | < 1.5 | < 1.5 | < 1.5 | **45** | < 1.5 | 5.8 | 1.7 |
| *ATF5* | *f* | < 1.5 | 2.1 | < 1.5 | **29** | < 1.5 | < 1.5 | < 1.3 | < 1.3 |

Numbers represent fold-increases of DEGs relative to the medium controls; numbers in bold represent the largest fold increase across the six cell models;

a. Nrf2-response; b. UPR-response; c. p53-signalling; d. Ferroptosis; e. Nuclear receptor Meta-pathway; f. Response to heme deficiency; g. Mitochondrial stress response.

*Elkin et al. 2021
